# Neddylation is a novel therapeutic target for lupus by regulating double negative T cell homeostasis

**DOI:** 10.1038/s41392-023-01709-9

**Published:** 2024-01-15

**Authors:** Yun Zhang, Lijun Du, Chenxi Wang, Zhangsheng Jiang, Qingchi Duan, Yiping Li, Zhijun Xie, Zhixing He, Yi Sun, Lin Huang, Liwei Lu, Chengping Wen

**Affiliations:** 1 Key Laboratory of Chinese medicine rheumatology of Zhejiang Province, Research Institute of Chinese Medical Clinical Foundation and Immunology, College of Basic Medical Science, Zhejiang Chinese Medical University, Hangzhou, 310053 China; 2 Department of General Surgery, Sir Run Run Shaw Hospital, Zhejiang University School of Medicine, Hangzhou, 310016 China; 3 Cancer Institute (Key Laboratory of Cancer Prevention and Intervention, China National Ministry of Education) of the Second Affiliated Hospital and Institute of Translational Medicine, Zhejiang University School of Medicine, Hangzhou, 310029 China; 4 Cancer Center of Zhejiang University, Hangzhou, 310029 China; 5 Department of Pathology and Shenzhen Institute of Research and Innovation, The University of Hong Kong, Hong Kong, China; 6 Chongqing International Institute for Immunology, Chongqing, 400038 China

**Keywords:** Rheumatic diseases, Immunological disorders

## Abstract

Systemic lupus erythematosus (SLE), a severe autoimmune disorder, is characterized by systemic inflammatory response, autoantibody accumulation and damage to organs. The dysregulation of double-negative (DN) T cells is considered as a crucial commander during SLE. Neddylation, a significant type of protein post-translational modification (PTM), has been well-proved to regulate T cell-mediated immune response. However, the function of neddylation in SLE is still unknown. Here, we reported that neddylation inactivation with MLN4924, a specific inhibitor of NEDD8-activating enzyme E1 (NAE1), or genetic abrogation of Ube2m in T cells decreased DN T cell accumulation and attenuated murine lupus development. Further investigations revealed that inactivation of neddylation blocked Bim ubiquitination degradation and maintained Bim level in DN T cells, contributing to the apoptosis of the accumulated DN T cells in lupus mice. Then double knockout (KO) lupus-prone mice (*Ube2m*^*-/-*^*Bim*^*-/-*^*lpr*) were generated and results showed that loss of Bim reduced Ube2m deficiency-induced apoptosis in DN T cells and reversed the alleviated lupus progression. Our findings identified that neddylation inactivation promoted Bim-mediated DN T cell apoptosis and attenuated lupus progression. Clinically, we also found that in SLE patients, the proportion of DN T cells was raised and their apoptosis was reduced. Moreover, compared to healthy groups, SLE patients exhibited decreased Bim levels and elevated Cullin1 neddylation levels. Meantime, the inhibition of neddylation induced Bim-dependent apoptosis of DN T cells isolated from SLE patients. Altogether, our findings provide the direct evidence about the function of neddylation during lupus, suggesting a promising therapeutic approach for this disease.

## Introduction

Systemic lupus erythematosus (SLE) is a multisystem autoimmune disorder with the breakdown in self-tolerance and the generation of autoantibodies.^1–3^ Double-negative (CD3^+^CD4^-^CD8^-^, DN) T cells, a unique subset of T cells lacking CD4 and CD8 co-receptors, play a significant role in the pathogenesis of autoimmune diseases, such as SLE.^4^ During SLE progress, DN T cells invade into multiple organs, contributing to the loss of tolerance. Besides, DN T cells are able to facilitate B cell differentiation to enhance the generation of autoantibodies. Moreover, DN T cells produce significant amounts of IFN-γ and IL-17, which can promote the development of SLE.^5–7^ As a result, the homeostasis of DN T cells is critical for lupus pathogenesis.^6,8–10^ Thus, elucidation of the signaling events mediating the homeostasis of DN T cells could provide new potential therapeutic options for SLE.

Neddylation is a type of protein post-translational modification (PTM). In this process, neuronal precursor cell-expressed developmentally downregulated protein 8 (NEDD8), an ubiquitin-like protein, binds to substrate proteins to regulate their stability, localization or activity.^11,12^ Similar to ubiquitination, neddylation is also catalyzed by a three-step enzymatic cascade of NEDD8-activating enzyme E1 (NAE1), NEDD8-conjugating enzyme E2 (Ube2m or Ube2f) and substrate-specific NEDD8-E3 ligases (RBX1/ROC1, RBX2/ROC2, etc.).^13^ The Cullin family, indispensable parts of Cullin-RING ubiquitin E3 ligases (CRL), is well-characterized as substrates for NEDD8 modification. Cullin neddylation is essential for the ubiquitin ligase activity of CRLs, which transfer the ubiquitin from recruited E2-ubiquitin to protein substrates and finally promote substrate ubiquitination.^14,15^

It is well established that neddylation modification is a critical PTM in modulating T cell-mediated immune response. Recently published researches have demonstrated the essential role of neddylation in regulatory T cell fitness, as well as its requirement for CD4^+^ T cell activation, survival, proliferation and T cell differentiation into various T helper subsets (Th1, Th2), regulatory T (Treg) cells, and T follicular helper (Tfh) cells to effectively regulate immune-related disorders.^16–19^ Thus, neddylation serves as a critical modulator for T cell functions. However, the precise involvement of neddylation in SLE remains unclear.

In our study, we firstly identified that blockade of neddylation pathway with MLN4924, a pharmacological inhibitor of NAE,^20^ significantly attenuated SLE progress with reduced DN T cell number in lupus-prone mice. To explore the function of neddylation pathway in SLE, Ube2m, the NEDD8-conjugating enzyme E2, was specifically knockout (KO) to generate spontaneous lupus-prone mice with Ube2m deficiency in T cells (*Ube2m*^*-/-*^*lpr*), where the neddylation was inactivated. Results showed that Ube2m deficiency attenuated SLE development. Subsequent experiments revealed that this effect resulted from the decreased number of DN T cells. Mechanism studies identified that inactivation of neddylation impaired Bim ubiquitination degradation and maintained Bim level in DN T cells, which induced the apoptosis of accumulated DN T cells in lupus mice. To confirm that neddylation pathway regulated the homeostasis of DN T cells via Bim, we generated the double KO lupus-prone mice (*Ube2m*^*-/-*^*Bim*^*-/-*^*lpr*) and found that the deficiency of Bim disrupted the apoptosis of DN T cells for Ube2m deficiency and cannot inhibit the development of lupus. Clinically, we also found that SLE patients exhibited an accumulation of DN T cells with reduced apoptosis. Furthermore, comparing the DN T cells from SLE groups with the healthy control, we discovered that the neddylation level of Cullin1 was enhanced while Bim level was decreased. Further research showed that neddylation inhibition with MLN4924 facilitated Bim-dependent apoptosis in DN T cells isolated from SLE groups. Our results confirmed that neddylation is necessary for the homeostasis of DN T cells. Neddylation inhibition initiates Bim-mediated mitochondrial apoptosis and restores the disordered immune tolerance in SLE. These results highlight the importance of neddylation pathway for SLE and suggest a novel therapeutic intervention of lupus via targeting neddylation pathway in DN T cells.

## Results

### Inhibition of neddylation attenuated lupus progression in MRL*/lpr* mice

**Fig. 1:**
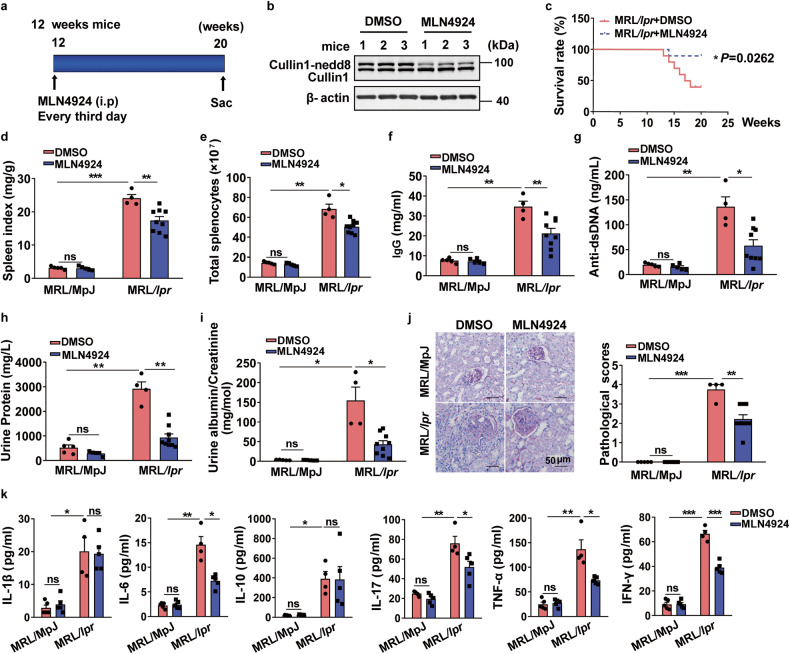
MLN4924 significantly attenuated SLE progress of MRL/*lpr* mice. **a** The female MRL/*lpr* and MRL/MpJ mice (12 weeks) were randomly allocated into two groups and treated with either control or MLN4924 (10 mg/kg) following the indicated scheme. *n* = 5 (MRL/MpJ group) or *n* = 10 (MRL*/lpr* group). **b** The inhibition of neddylation with MLN4924 was evaluated via the neddylation level of Cullin1 in splenocytes. **c** Survival curve of MRL/*lpr* mice treated with control or MLN4924. *n* = 10 mice/group. *P < 0.05. **d**, **e** Spleen index (spleen weight /body weight ratio) and the number of splenocytes were calculated. *n* = 5 (MRL/MpJ group), 4 (MRL/*lpr* treated with DMSO), or 9 (MRL/*lpr* treated with MLN4924). *P < 0.05, **P < 0.01, ***P < 0.001. **f**, **g** The serum was collected at 20 weeks, followed by monitoring of IgG and anti-dsDNA antibody levels. *n* = 5 (MRL/MpJ group), 4 (MRL/*lpr* administered with DMSO), or 9 (MRL/*lpr* administered with MLN4924). *P < 0.05, **P < 0.01. **h**, **i** Total protein, albumin and creatinine in urine were assessed and albumin/creatinine was calculated. *n* = 5 (MRL/MpJ group), 4 (MRL/*lpr* administered with DMSO), or 9 (MRL*/lpr* administered with MLN4924). *P < 0.05, **P < 0.01. **j** Representative images of PAS staining of kidneys from 20-week-old mice. Scale bar = 50 µm. Then, pathological score was calculated. *n* = 5 (MRL/MpJ group), 4 (MRL/*lpr* administered with DMSO), or 9 (MRL/*lpr* administered with MLN4924). **P < 0.01, ***P < 0.001. **k** Cytokine profile in serum was analyzed using Bio-Plex Pro^TM^ Mouse Cytokine Th17 panel A6-plex. *n* = 5 (MRL/MpJ groups, MRL/*lpr* administered with MLN4924 group) or 4 (MRL/*lpr* administered with DMSO). *P < 0.05, **P < 0.01, ***P < 0.001

To investigate the function of neddylation during lupus, female MRL/*lpr* mice with the *Fas* gene mutation were used. From about 12 weeks of age, MRL/*lpr* mice exhibit the expansion of lymphocytes and spontaneously develop SLE-like symptoms resembling human SLE.^10^ We then treated MRL/MpJ and MRL/*lpr* group with DMSO or MLN4924 (a specific inhibitor of neddylation) every 3rd day from 12 weeks to 20 weeks of age (Fig. 1a). Firstly, we found that treatment of MLN4924 remarkably inhibited the neddylation of Cullin1 in splenocytes of MRL/*lpr* mice (Fig. 1b), suggesting that MLN4924 indeed suppressed neddylation pathway in vivo. Notably, MRL/*lpr* mice treated with MLN4924 showed significantly higher survival rates than controls (Fig. 1c). Besides, MLN4924 treatment resulted in a significant reduction in spleen size as evidenced by a decreased spleen index (spleen weight/body weight ratio) (Fig. 1d) and decreased splenocyte number in MRL/*lpr* mice (Fig. 1e). Additionally, we assessed the levels of serum IgG and anti-dsDNA antibodies, which are important diagnostic markers of lupus.^21^ We found that MLN4924 prominently reduced IgG (Fig. 1f) and anti-dsDNA (Fig. 1g) antibody levels in MRL/*lpr* group (Fig. 1f, g). To assess the impact of MLN4924 on kidney function, urine samples were obtained and the levels of urinary total protein, albumin and creatinine were quantified. We noticed that MRL/*lpr* mice administrated with MLN4924 exhibited a significantly decrease in total protein (Fig. 1h) and albumin/creatinine ratio (Fig. 1i). Histological analysis of kidney sections stained with PAS revealed that MLN4924 markedly reduced the crescent glomerulonephritis in MRL/*lpr* group (Fig. 1j), indicating a protective role of MLN4924 in renal function. Serum cytokine levels were evaluated and results indicated that the production of IL-6, IL-17, TNF-α and IFN-γ was inhibited in MRL/*lpr* group with MLN4924 administration while IL-1β and IL-10 level remained unchanged (Fig. 1k). Together, our data suggest that MLN4924 treatment attenuates lupus symptoms as exemplified by increased survival rate, reduced splenomegaly and autoantibody production, ameliorated renal function, and suppressed inflammatory cytokine levels.

### MLN4924 significantly reduced the number of DN T cells in MRL/*lpr* mice

**Fig. 2:**
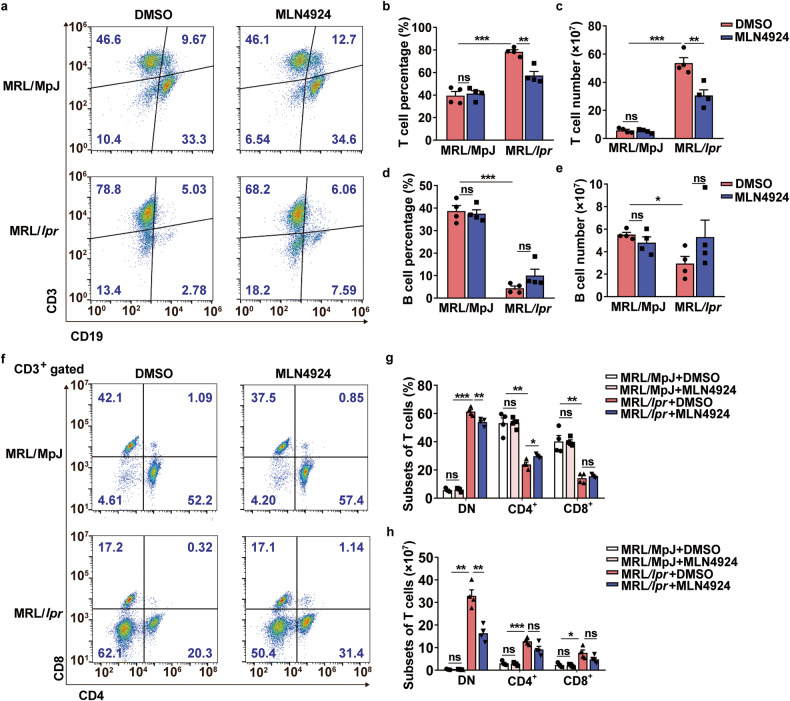
Reduced T cell accumulation in MLN4924-treated MRL*/lpr* mice. **a** Proportion of T and B cells in spleens was evaluated by flow cytometry. *n* = 4/group. **b**–**e** The percentage and absolute numbers of T cells and B cells were quantified and calculated statistically based on the analysis of flow cytometry. *n* = 4/group. *P < 0.05, **P < 0.01, ***P < 0.001. **f** CD3^+^ T cell subsets in spleens were analyzed using flow cytometry. *n* = 4/group. **g**, **h** Percentage and total count of T cell subsets were quantified and calculated based on the flow cytometric analysis, *n* = 4/group. *P < 0.05, **P < 0.01, ***P < 0.001

As indicated in Fig. 1e, splenic cell accumulation was considerably reduced for MLN4924 treatment. Thus, we investigated the impact of MLN4924 on splenic cells by examining the T and B cell proportions. Flow cytometry analysis revealed a significant decrease of the percentage and total count of T cells in MLN4924-administered MRL/*lpr* mice compared to DMSO-treated mice (Fig. 2a–c) while the proportion and number of B cells remained unchanged (Fig. 2a, d, e). Further analysis showed a moderate reduction in DN T cell percentage but a significant decrease in DN T cell number for MLN4924 treatment (Fig. 2f–h), indicating that inhibition of neddylation suppressed the accumulation of DN T cells in MRL/*lpr* mice.

### Deficiency of Ube2m in T cells attenuated SLE development

To thoroughly assess the role of neddylation in SLE, Lckcre*Ube2m*^*fl/fl*^MRL.*Fas*^*lpr*^ mice (termed *Ube2m*^*-/-*^*lpr* mice) were generated according to the procedure in Supplementary Fig. 1a. *Ube2m*^*-/-*^*lpr* mice showed Ube2m-specific deletion in T cells and spontaneously developed lupus. To assess deletion efficiency, Ube2m protein levels were monitored and results showed a clear reduction in purified T cells of *Ube2m*^*-/-*^*lpr* mice, while Ube2f, another E2 NEDD8 conjugating enzyme, remains unchanged (Supplementary Fig. 1b), which suggested the specific deletion of Ube2m in *Ube2m*^*-/-*^ mice.

**Fig. 3:**
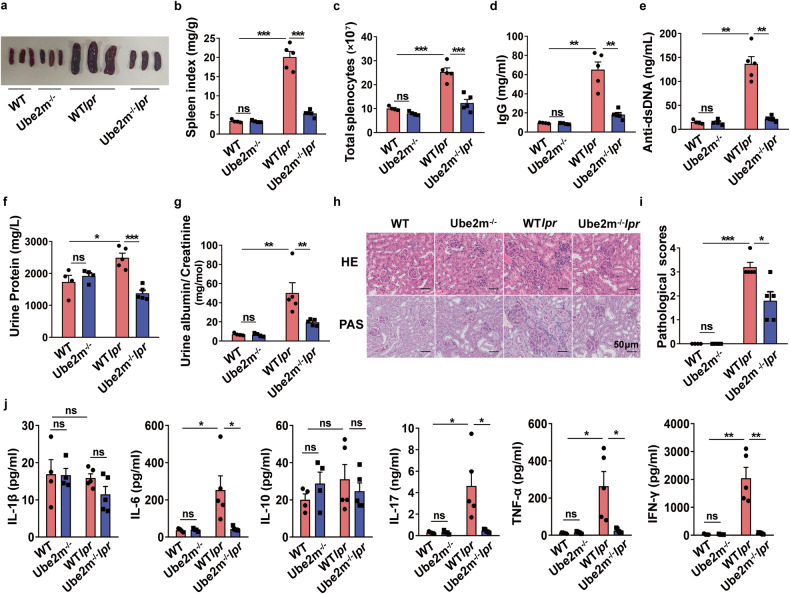
Ube2m-deficient mice were protected from lupus. **a** Representative photographs of spleens from WT, Ube2m^-/-^, WT*lpr* and Ube2m^-/-^*lpr* mice at 8 months. **b**, **c** Spleen index and the number of splenocytes were calculated. *n* = 4 or 5/group. ***P < 0.001. **d**, **e** The serum was collected at 8 months then level of IgG and anti-dsDNA antibodies were measured. *n* = 4 or 5/group. **P < 0.01. **f**, **g** Total protein, albumin and creatinine in urine were assessed and albumin/creatinine was calculated. *n* = 4 or 5/group. *P < 0.05, **P < 0.01, ***P < 0.001. **h**, **i** Representative images of PAS and HE staining from kidneys of 8-month-old mice were shown. Scale bar = 50 µm. Then, pathological score was calculated. *n* = 4 or 5/group. *P < 0.05, ***P < 0.001. **j** Cytokine profile in serum was measured using Bio-Plex Pro^TM^ Mouse Cytokine Th17 panel A6-plex. *n* = 4 or 5/group. *P < 0.05, **P < 0.01

Both the percentages and numbers of different T cell subsets from thymus of these mice were analyzed and results showed that *Ube2m*^*-/-*^ and *Ube2m*^*-/-*^*lpr* mice exhibited normal T cell development in thymus, including normal DN T cells, DP cells, CD4^+^ and CD8^+^ T cells (Supplementary Fig. 2a–e). Based on the presence of the specific surface markers, CD25 and CD44, the DN stages have been classified as DN1, DN2, DN3 and DN4.^22^ Therefore, we further characterized DN populations according to the expression of CD25 and CD44 to explore whether Ube2m deficiency in T cells affects the development of DN T cells. As shown in Supplementary Fig. 2c, f, g, T cells lacking Ube2m exhibited unchanged DN T cells. The normal development of T cells in thymus of *Ube2m* KO mice allowed further investigation of the effects of Ube2m deficiency on the pathogenesis of lupus. As shown in Fig. 3a–c, *Ube2m*^*-/-*^*lpr* mice showed dramatically reduced splenomegaly (Fig. 3a, b) and decreased number of splenocytes (Fig. 3c) compared with WT*lpr* at 8 months. Moreover, the serum IgG and anti-dsDNA antibody production was notably abated (Fig. 3d, e). Furthermore, we also found that deficiency of Ube2m ameliorated lupus nephritis, including lessened total protein (Fig. 3f) and albumin/ creatinine ratio (Fig. 3g), restored renal structures (Fig. 3h, i). In addition, *Ube2m*^*-/-*^*lpr* mice showed markedly reduced levels of inflammatory cytokines (Fig. 3j). Collectively, these results clearly showed that neddylation inactivation in T cells significantly attenuated lupus progression.

### Loss of Ube2m promoted DN T cell apoptosis

**Fig. 4:**
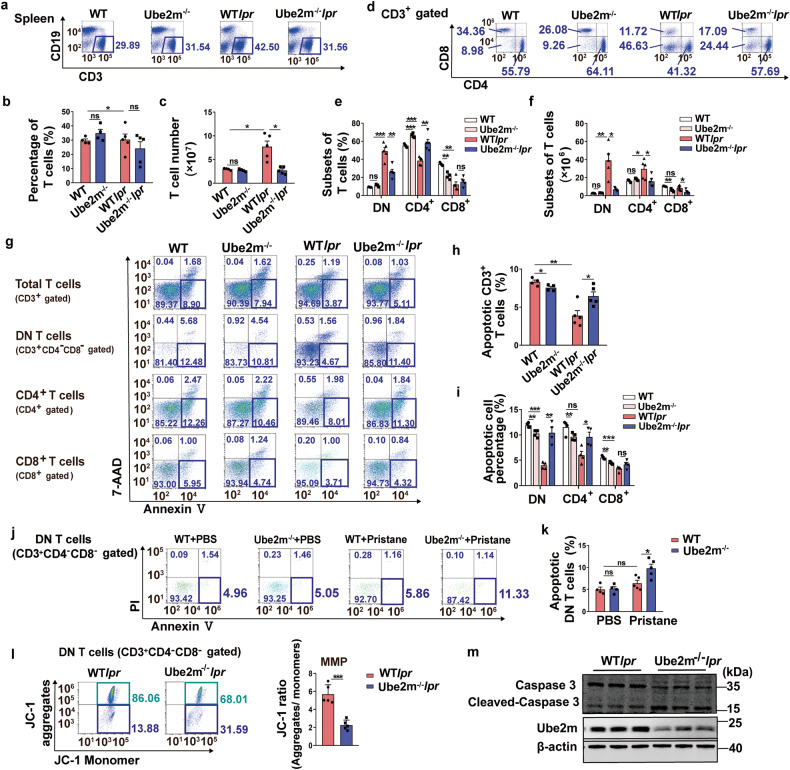
Increased DN T cells apoptosis in Ube2m KO lupus-prone mice. **a** Proportion of T cells in spleens was analyzed by flow cytometry. *n* = 4 or 5/group. **b**, **c** The percentage and number of T cells were calculated based on the data of flow cytometry. *n* = 4 or 5/group. *P < 0.05. **d** CD3^+^ T cell subsets of spleens were analyzed via flow cytometry. *n* = 4 or 5/group. **e**, **f** The percentage and count of T cell subsets were determined using flow cytometry data. *n* = 4 or 5/group. *P < 0.05, **P < 0.01, ***P < 0.001. **g** The early apoptosis (Annexin V^+^/7-AAD^-^) of total T cells, DN T cells, CD4^+^ and CD8^+^ T cells was evaluated with flow cytometry. *n* = 4 or 5/group. **h**, **i** The apoptosis of total T cells and T cell subsets was quantified based on the analysis of flow cytometry. n = 4 or 5/group. *P < 0.05, **P < 0.01, ***P < 0.001. **j** DN T cell apoptosis (Annexin V^+^/PI^-^) in spleens from pristine-induced mice was analyzed with flow cytometry. *n* = 4 or 5/group. **k** DN T cell apoptosis in spleens of pristine-induced mice was quantified based on the analysis of flow cytometry. *n* = 4 or 5/group. *P < 0.05. **l** MMP was assessed using JC-1 assay to indicate the apoptosis of DN T cells. *n* = 5/group. ***P < 0.001. **m** Cleaved-caspase 3 was detected via immunoblotting assay to indicate the apoptosis of DN T cells. *n* = 3/group

In vivo evidence establishing the crucial role of Ube2m in lupus development, we further explored how Ube2m regulated lupus progress. Firstly, we measured the number and proportion of T cells in spleen and results revealed that loss of Ube2m prominently blocked total T cell accumulation both in spleen (Fig. 4a–c) and peripheral blood (Supplementary Fig. 3a, b). Further research showed that DN T cell accumulation in spleen (Fig. 4d–f) and peripheral blood (Supplementary Fig. 3c, d) were significantly inhibited in *Ube2m*^*-/-*^*lpr*. Our previous results (Supplementary Fig. 2c–g) have demonstrated that the development of DN T cells in thymus is not affected by Ube2m KO. Therefore, we then detected the influence of Ube2m on DN T cells in spleens. In view of the deficit of Fas-triggered apoptosis in *lpr* mice,^23^ we firstly measured the level of T cell apoptosis in spleens. Consistent with our above-mentioned phenotype, the apoptosis of DN T cells was blocked in WT*lpr* mice compared to WT mice, while disruption of Ube2m restored the normal apoptosis of T cells, especially DN T cells (Fig. 4g–i). Similar results were found in peripheral blood (Supplementary Fig. 4a–c). Consistently, we also observed that loss of Ube2m significantly promoted DN T cell apoptosis (Fig. 4j, k) and attenuated disease progression (Supplementary Fig. 5) in pristane-induced lupus mice. Then, JC-1 staining assay indicated that Ube2m-deleted DN T cells exhibited reduced MMP, as evaluated by the ratio of JC-1 aggregates to monomer (Fig. 4l), which indicated increased apoptosis. Consistently, we also found that Ube2m deficiency resulted in an elevated cleaved-caspase 3 level (Fig. 4m) in DN T cells, which is required for cell apoptosis. Besides, the excessive T cell proliferation is another striking abnormality of *lpr* mice^24^ and our results showed that Ube2m deficiency inhibited total T cell proliferation in vivo (Supplementary Fig. 6a, b). Notably, loss of Ube2m mainly restrained proliferation of CD4^+^ and CD8^+^ T cells while having minimal effect on DN T cell proliferation (Supplementary Fig. 6a, c), which suggested differential functions of Ube2m in different T cell subsets. These findings demonstrated the essential role of Ube2m in the survival of DN T cells, and deletion of Ube2m facilitated DN T cell apoptosis in lupus mice.

### Neddylation inactivation upregulated Bim level by inhibiting Bim ubiquitination degradation

**Fig. 5:**
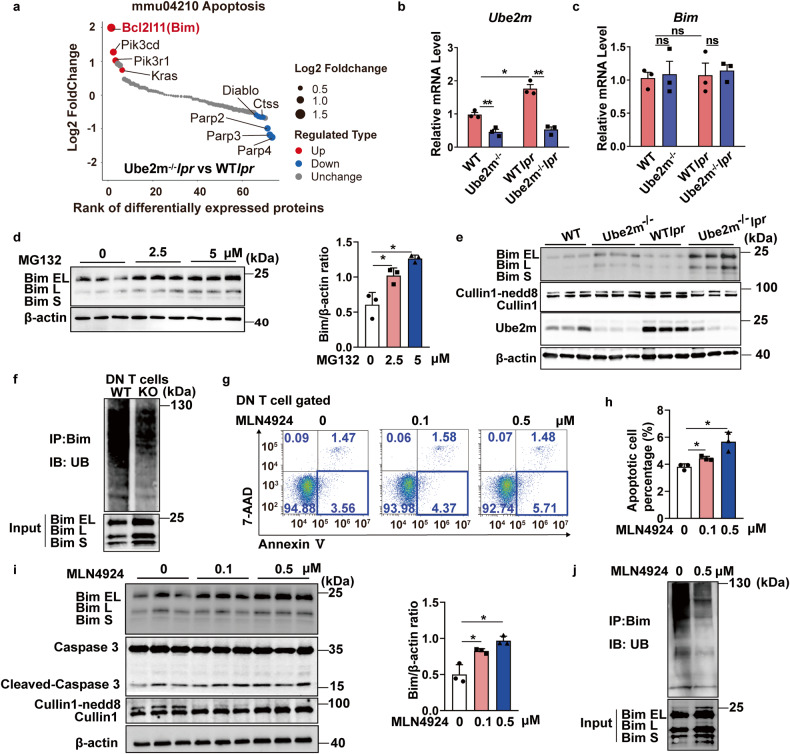
Bim level was increased with impaired ubiquitination degradation in DN T cells for neddylation inhibition. **a** The proteins annotated with apoptosis were selected and ranked based on the ratio value from the proteomic results of DN T cells. Red dots represented proteins significantly upregulated (FC＞ 1.5, *P* < 0.05); Blue dots represented proteins significantly downregulated (FC < 0.667, *P* < 0.05). *n* = 4/group. **b**, **c** The mRNA levels of Ube2m and Bim in DN T cells were evaluated via real-time PCR. *n* = 3/group. *P < 0.05, **P < 0.01. **d** Immunoblotting assay and densitometric analysis evaluated the protein level of Bim in DN T cells treated with MG-132. *P < 0.05. **e** Western blot assay was conducted to determine the level of Cullin1 neddylation, Ube2m and Bim in DN T cells. One band represented one mouse. *n* = 3. **f** Ubiquitination degradation of Bim in DN T cells was measured via Co-IP assay. Data were representative of three independent experiments. **g** The apoptosis of DN T cells treated with MLN4924 were detected via flow cytometry (Annexin V^+^/7-AAD^-^). **h** The apoptosis of DN T cells treated with MLN4924 was quantified based on the analysis of flow cytometry. *n* = 3/group. *P < 0.05. **i** Immunoblotting assay and densitometric analysis showed the level of Bim and Cleaved-caspase3 in MLN4924 treated-DN T cells. *P < 0.05. **j** Ubiquitination degradation of Bim in MLN4924-treated DN T cells was measured via Co-IP assay. Data were representative of three independent experiments

We have shown that loss of Ube2m promoted DN T cell apoptosis and contributed to alleviated lupus development (Figs. 3 and 4), while the mechanism remained unknown. Therefore, DN T cells were sorted and an unbiased quantitative proteomic analysis was conducted to screen for the differential protein between WT*lpr* and *Ube2m*^*-/-*^*lpr* mice. Results indicated that the protein Bim (encoded by the *Bcl2l11* gene) showed the most significant increase among the apoptosis-related proteins for Ube2m deficiency (Fig. 5a). Next, the mRNA level of Bim was measured and results indicated that the transcriptional expression of Bim remained unchanged for loss of Ube2m (Fig. 5b, c). Based on our data, we speculated that neddylation pathway regulated Bim degradation. Consequently, MG-132, a proteasome inhibitor, was used to treat DN T cells. Results indicated that Bim was accumulated upon MG-132 treatment (Fig. 5d), suggesting that Bim degradation was proteasome-dependent. Previous studies have proven that Bim ubiquitination degradation is dependent on the neddylation of Cullin1.^25,26^ As a result, the neddylation of Cullin1 was detected and results showed that Cullin1 neddylation was at a lower level in normal mice and remained little change for Ube2m deficiency, resulting in unchanged Bim protein level in DN T cells under normal physiological condition (Fig. 5e). However, we found that Ube2m expression and the neddylation of Cullin1 was significantly increased in lupus conditions (Fig. 5e), suggesting that the neddylation pathway activation was necessary for DN T cell abnormal survival in lupus progression. Then, loss of Ube2m prominently blocked the neddylation of Cullin1, contributing to the Bim protein accumulation (Fig. 5e) and finally promoting DN T cell apoptosis in lupus mice (Fig. 4g, i). Furthermore, Co-IP assay revealed that the ubiquitination degradation of Bim was indeed remarkably inhibited for Ube2m deletion in DN T cells (Fig. 5f). In vitro results also showed that neddylation inactivation with MLN4924 promoted DN T cell apoptosis (Fig. 5g, h), with elevated Bim and cleaved-caspase 3 protein levels (Fig. 5i). Further Co-IP assay also indicated that MLN4924 treatment disrupted Bim ubiquitination degradation, contributing to the accumulation of Bim (Fig. 5j). Taken together, we demonstrated that neddylation inactivation impairs Bim ubiquitination degradation and maintains Bim level, ultimately promoting the apoptosis of DN T cells in lupus mice.

### Loss of Bim reduced Ube2m deficiency-induced apoptosis in DN T cells and reversed the alleviated lupus progression

**Fig. 6:**
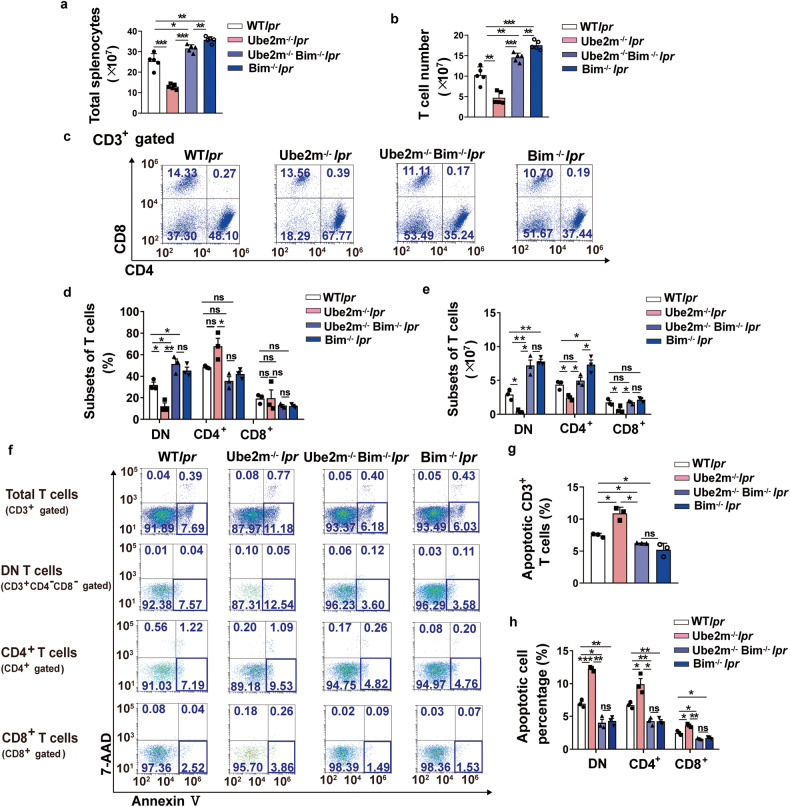
Loss of Bim reduced Ube2m deletion-induced apoptosis of DN T cells. **a** The number of splenocytes was calculated. *n* = 5/group. *P < 0.05, **P < 0.01, ***P < 0.001. **b** The number of T cells in spleens was measured. *n* = 5/group. **P < 0.01, ***P < 0.001. **c** CD3^+^ T cell subsets in spleens were analyzed using flow cytometry. *n* = 3/group. **d**, **e** The percentage and number of T cell subsets were quantified and calculated based on the flow cytometry data. *n* = 3/group. *P < 0.05, **P < 0.01. **f**–**h** The apoptosis (Annexin V^+^/7-AAD^-^) of total T cells, DN T cells, CD4^+^ and CD8^+^ T cells was evaluated and quantified. *n* = 3/group. *P < 0.05, **P < 0.01, ***P < 0.001

According to our obtained findings, we hypothesized that Bim is a pivotal protein downstream mediated by neddylation pathway, responsible for DN T cell homeostasis. To further test our hypothesis, Lckcre*Bim*^*fl/fl*^MRL.*Fas*^*lpr*^ (termed *Bim*^*-/-*^*lpr*) and Lckcre*Ube2m*^*fl/fl*^*Bim*^*fl/fl*^MRL.*Fas*^*lpr*^ (termed *Ube2m*^*-/-*^*Bim*^*-/-*^*lpr*) mice were generated. Firstly, we found that deletion of Bim in T cells led to an increase both in splenic and T cell numbers (Fig. 6a, b). Further analysis revealed that there was a dramatic increase in the proportion and number of DN T cells (Fig. 6c–e) due to reduced apoptosis (Fig. 6f–h) in *Bim*^*-/-*^*lpr* compared with WT*lpr* mice, suggesting a significant function of Bim in DN T cell homeostasis. Then, we also found that lack of Bim blocked the increased apoptosis of DN T cells resulted from Ube2m deficiency (Fig. 6f, h), contributing to more DN T cells in *Ube2m*^*-/-*^*Bim*^*-/-*^*lpr* mice compared with *Ube2m*^*-/-*^*lpr* mice (Fig. 6c–e). Moreover, when Bim is lost, the lack of Ube2m has little effects on the apoptosis and number of DN T cells (Fig. 6c–h). Thus, Bim served as a key downstream protein of neddylation pathway to regulate DN T cell apoptosis. In addition, *Ube2m*^*-/-*^*Bim*^*-/-*^*lpr* mice showed reduced T cell (Fig. 6b) and CD4^+^ T cell number (Fig. 6e) with unchanged T cell and CD4^+^ T cell apoptosis compared with *Bim*^*-/-*^*lpr* mice (Fig. 6f–h), which suggested that there are some Bim-independent mechanisms to regulate CD4^+^ T cell homeostasis.

Considering the important function of DN T cells in lupus progress, lupus syndrome of these mice was also detected in Supplementary Fig. 7. Consistently, *Bim*^*-/-*^*lpr* mice exhibited the most severe lupus syndrome, including splenomegaly (Supplementary Fig. 7a), the accumulation of autoantibodies (Supplementary Fig. 7b, c), renal destruction (Supplementary Fig. 7d–g) as well as increased inflammatory cytokine level (Supplementary Fig. 7 h). Meanwhile, compared with *Bim*^*-/-*^*lpr* mice, *Ube2m*^*-/-*^*Bim*^*-/-*^*lpr* mice also showed slightly remission on lupus development (Supplementary Fig. 7), indicating a Bim-independent manner of Ube2m on lupus development. In addition, we also found that the attenuated lupus phenotypes in Ube2m KO mice were mainly reversed in *Ube2m*^*-/-*^*Bim*^*-/-*^*lpr* mice (Supplementary Fig. 7). Collectively, these evidences clearly identified that Bim is an indispensable downstream protein governed by neddylation pathway to regulate DN T cell apoptosis, which mediates the development of lupus.

### Inhibition of neddylation pathway promoted Bim-dependent DN T cell apoptosis in SLE patients

**Fig. 7:**
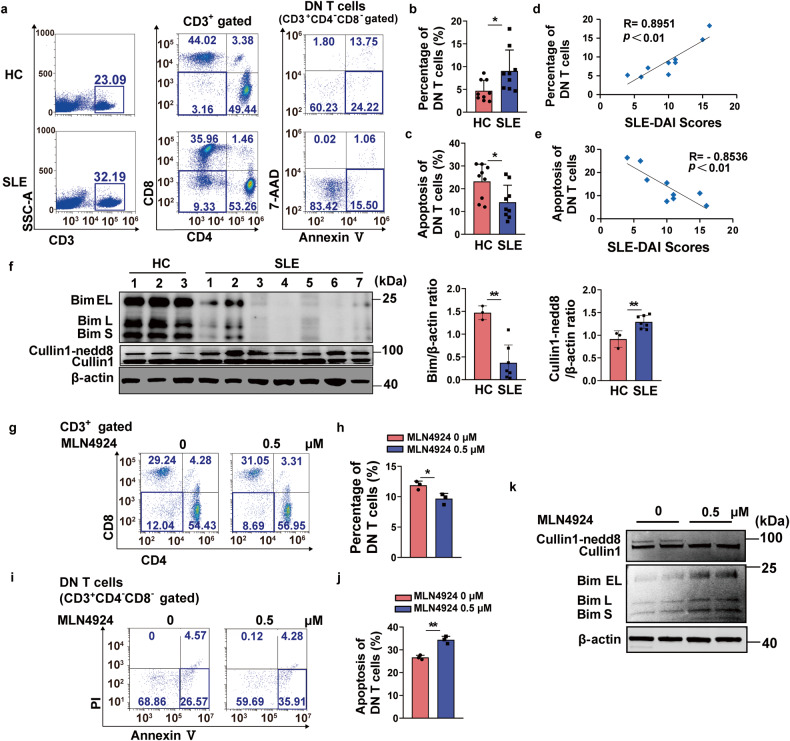
Neddylation inhibition induced the Bim-dependent DN T cell apoptosis isolated from SLE patients. **a** The percentage and the apoptosis of DN T cells from both SLE groups and healthy individuals were assessed using flow cytometry. *n* = 9/group. **b** DN T cell percentage was quantified based on the results of flow cytometry. *n* = 9/group. *P < 0.05. **c** The proportion of DN T cell apoptosis were quantified based on the results of flow cytometry. *n* = 9/group. *P < 0.05. **d** Analysis of the correlation between the SLE-DAI scores and the percentage of DN T cells in SLE groups. *n* = 9/group. *R* = 0.8951, **P < 0.01. **e** Analysis of the correlation between the SLE-DAI scores and the apoptosis percentage of DN T cells in SLE groups. *n* = 9/group. *R* = −0.8536, **P < 0.01. **f** Immunoblotting assay and densitometric analysis evaluated the protein level of Bim and Cullin1 neddylation in DN T cells isolated from healthy and SLE group. In HC group, each band was a combination of two samples, while in SLE group, one band represented one samples. **P < 0.01. **g**, **h** The PBMC isolated from SLE patients was incubated with MLN4924 (0.5 μM) and the proportion of DN T cells were analyzed via flow cytometry. The results were quantified based on the data of flow cytometry. *n* = 3/group. *P < 0.05. **i**, **j** The PBMC isolated from SLE patients was treated with MLN4924 (0.5 μM) and the apoptosis of DN T cells were assessed via flow cytometry (Annexin V^+^/PI ^-^). The results were quantified based on the flow cytometry data. *n* = 3/group. **P < 0.01. **k** Immunoblotting assay evaluated the protein level of Bim and Cullin1 neddylation in DN T cells isolated from SLE patients treated with MLN4924 (0.5 μM). Data were representative of three independent experiments

We have verified that neddylation inactivation induced Bim-dependent DN T cell apoptosis in lupus-prone mice. However, the impact of neddylation inactivation on DN T cells derived from SLE patients remains unknown. Peripheral blood samples were collected from both healthy individuals and patients with active SLE. Firstly, we observed that patients with SLE showed a higher percentage of total DN T cells with lower apoptosis in comparison to the healthy group (Fig. 7a–c). The subsequent correlation analysis revealed a positive association between the percentage of DN T cells and SLE-DAI scores (Fig. 7d), then a negative association between the apoptosis proportion of DN T cells and SLE-DAI scores (Fig. 7e). Further exploration showed that Bim protein level was reduced, while the neddylation of Cullin1 was increased in DN T cells of SLE group (Fig. 7f). On this basis, MLN4924 was used to treat the PBMC isolated from SLE patients and results revealed that neddylation inhibition declined the DN T cell percentage (Fig. 7g, h) and promoted the apoptosis of DN T cells (Fig. 7i, j). Consistently, we found the Bim level was upregulated (Fig. 7k) upon neddylation inhibition. These clinical data identified that neddylation inactivation promotes the Bim-dependent DN T cell apoptosis, thus contributing to the reduction of DN T cells. Our data suggested that inhibition of neddylation pathway is a promising therapeutic option for SLE.

**Fig. 8:**
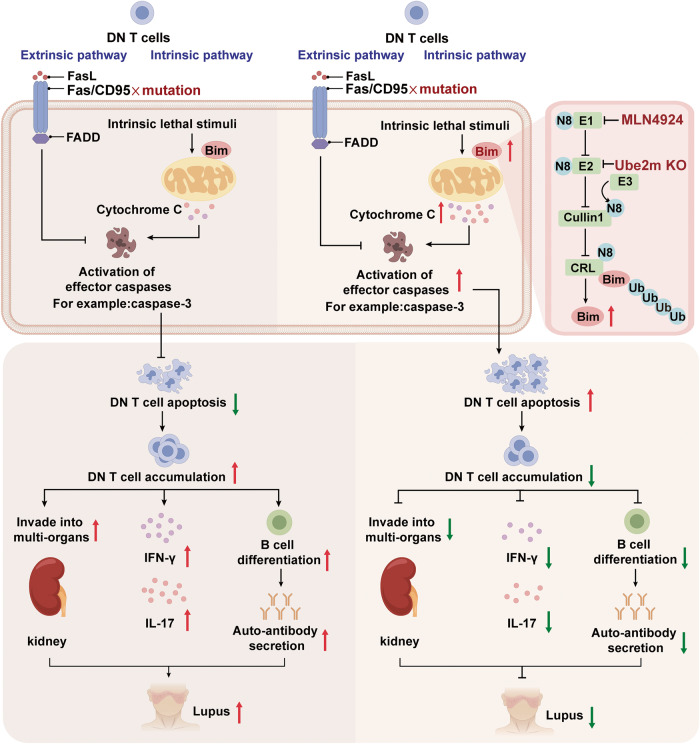
Schematic illustration showed the mechanism of neddylation pathway in lupus treatment. DN T cells enrichment results in the destruction of immune tolerance with the mass secretion of IFN-γ and IL-17, autoantibody accumulation and the damage of multi-organs, leading to the development of lupus. Inactivation of neddylation pathway (MLN4924 treatment or Ube2m KO in T cells) impaired the neddylation of Cullin1 and then interrupted the ubiquitination of Bim, contributing to the Bim-dependent DN T cell apoptosis, which restores the immune tolerance and finally alleviates lupus symptoms. This figure was created by Figdraw (www.figdraw.com)

In conclusion, our research uncovers the role of neddylation pathway in DN T cell homeostasis (Fig. 8), thereby providing a novel treatment approach for SLE.

## Discussion

SLE is an autoimmune disorder and the underlying causes remains mostly unknown. The abnormal accumulation of DN T cells, which led to the peripheral tolerance defects and systemic damage, is a key pathogenesis of SLE.^4,27^ Thus, it is necessary to elucidate the mechanisms controlling the homeostasis of DN T cells during SLE. Here, we functionally characterized the unique role of neddylation in DN T cell homeostasis with a specific inhibitor of neddylation and a genetic approach. Neddylation inactivation promoted the apoptosis of DN T cells via stabilizing the Bim level and contributed to SLE remission. These findings underscore the crucial role of neddylation in DN T cell functions and indicate neddylation as a potential target for SLE therapy.

Neddylation, a post-translational modification, attaches NEDD8 to substrate proteins to affect their localization, stability or activity. CRLs can be activated by neddylation, which facilitate the ubiquitylation and degradation of substrates and then mediate lots of cellular processed including cell cycle progression, cell apoptosis and cell survival.^28–30^ In addition, ongoing investigations implicate neddylation is necessary for the regulation of immune cell functions and involved in the pathogenesis of related immune disorders.^31^ For example, neddylation plays a pivotal role in pro-inflammatory cytokine production in innate immune cells, including macrophages, neutrophils and dendritic cells (DCs).^32–34^ Neddylation is also required for the IFN production against RNA viral infection in myeloid DCs.^35^ Additionally, neddylation has been proven as an indispensable process for the activation, proliferation and polarization of CD4^+^ T cells to regulate T cell-mediated immunity response.^16,19,36–38^ However, the role of neddylation in DN T cells remains to be explored.

The homeostasis of DN T cells was involved in the development of SLE.^4,10^ DN T cell apoptosis and timely clearance are critical for immune tolerance, which avoided the release of autoantigen and the induction of autoimmunity. Once the apoptosis of DN T cell was disturbed, excessive accumulation of DN T cells will initiate autoimmune response.^4,39–41^ Therefore, the normal apoptosis and clearance of DN T cells are beneficial for lupus remission. Bim, belonging to the Bcl-2 family, is a crucial BH3 protein promoting cell intrinsic apoptosis.^42^ In mammalian, two main pathways govern the initiation of apoptosis. One is the extrinsic pathway (or death receptor pathway), triggered by ligand engagement of cell surface death receptors such as Fas. Another is intrinsic apoptotic pathway, which is mediated by Bcl-2 protein family.^43^ Fas-dependent death receptor pathway and Bim-mediated intrinsic apoptosis have been proven to suppress chronic immune responses and prevent autoimmunity.^44–46^ Loss of either Fas or Bim resulted in marked lymphadenopathy and splenomegaly, and deficiency of Bim in MRL*/lpr* mice developed more extreme lymphadenopathy, indicating that Fas signaling and Bim showed overlapping but non-redundant roles in lymphadenopathy and splenomegaly.^47,48^ However, the precise role of Bim in DN T cells and the underlying regulatory mechanism governing Bim homeostasis remained elusive. In our study, we found that neddylation inactivation observably reduced DN T cell accumulation in lupus mice with upregulated Bim protein level and normal transcription of Bim. MG-132 treatment suggested that Bim degradation was mediated by ubiquitin-proteasome system. Previous studies have proven that Bim protein was the substrates of CRL1 and neddylation inhibition increased Bim protein level to promote the apoptosis of B cells.^25,26,49^ Therefore, we also detected the Bim ubiquitination degradation and found that the Bim degradation was significantly inhibited accompanied with impaired Cullin1 neddylation in DN T cells when neddylation was inactivated. However, Bim accumulation and Cullin1 neddylation inhibition of DN T cells were only found in lupus mice rather than in non-lupus group. Our data indicated that Ube2m specifically regulates Bim in SLE group. In normal physiological condition, the remaining low level of Ube2m after Cre driven deletion appears to be sufficient to maintain the basal level of Cullin1 neddylation. However, under lupus conditions, Ube2m was significantly induced to ensure sufficient Cullin1 neddylation to activate CRL1, which is consistent with the previous work showing that Ube2m is a stress inducible protein.^50^ And Ube2m deletion failed to provide enough Cullin1 neddylation, leading to CRL1 inactivation and subsequent Bim accumulation to induce apoptosis in lupus groups. To further verify the involvement of Bim in neddylation-mediated DN T cell apoptosis, *Ube2m*^*-/-*^*Bim*^*-/-*^*lpr* mice were generated and results showed that when Bim was deleted, neddylation inactivation failed to promote DN T cell apoptosis, suggesting that neddylation pathway mediated DN T cell apoptosis via regulating Bim protein degradation.

In our research, we also found that neddylation function has heterogeneity in different T cell subsets. Our data revealed that neddylation inactivation also blocked Bim ubiquitination and increased Bim protein level in CD4^+^ and CD8^+^ T cells similar with DN T cells (Supplementary Fig. 8a–d). However, the susceptibility of DN T cells to Bim-induced apoptosis was found to be the highest (Fig. 4g–i, Fig. 5g, h and Supplementary Fig. 8e–h). It has been well demonstrated that cellular metabolism can influence cell sensitivity to apoptosis.^51,52^ We hypothesized that the heterogeneity of neddylation functions in different T cells depended on their distinct metabolic profiles. More research is necessary to comprehensively elucidate the metabolic modes of different T cells as well as the relationship between apoptosis and metabolic modes during lupus. Besides, we found that neddylation inactivation notably suppressed the proliferation of CD4^+^ T cells and blocked the differentiation of T follicular helper (Tfh) cells in MRL/*lpr* mice and pristane-induced lupus models (data not shown). CD4^+^ T cells, such as Th1, Tfh and Th17 cells also contribute to tissue inflammation and autoantibody production, promoting the pathogenesis of SLE.^2,53,54^ These changes also explained why *Ube2m*^*-/-*^*Bim*^*-/-*^*lpr* mice exhibited slightly reduced T cell number and attenuated lupus symptoms compared with *Bim*^*-/-*^*lpr* mice. However, the mechanism of neddylation pathway in regulating the function of CD4^+^ T cells during SLE progression requires further investigation.

In summary, we have identified a significant role of neddylation in maintaining DN T cell homeostasis and mediating SLE progression. Suppression of neddylation with MLN4924 or genetic abrogation of Ube2m significantly ameliorated the progression of SLE via reconstructing the normal apoptosis of DN T cells. Further experiments indicated that the interception of apoptosis in lupus mice was indeed recovered for neddylation inactivation, which resulted from the inhibition of Bim ubiquitination and the rise of Bim-mediated apoptosis. The clinical data also showed that SLE patients displayed accumulated DN T cells with defects in apoptosis while neddylation inhibition promoted the apoptosis of DN T cells via up-regulating Bim protein level. The findings enhance our comprehension about the pathogenesis of SLE and suggest that neddylation pathway might be a promising target for SLE therapy.

## Materials and methods

### Mice

We purchased MRL/*lpr* and the control MRL/Mpj mice (female, 6 weeks) from SLRC laboratory Animal centre. From 12 weeks to 20 weeks, MLN4924 (15 mg/kg) (MCE) or DMSO were intraperitoneally injected into these mice every 3rd day. The mice status and the deaths were observed and recorded daily. The mice were narcotized at 20 weeks in order to obtain serum and blood for flow cytometry. After that, the mice were sacrificed. Kidneys and spleens were obtained. The weight of spleens was evaluated and the total count of splenic cells was determined using the CEDEX XS system (Roche). The *Ube2m*^*fl/fl*^ mice were generous gifts from Yi Sun (Zhejiang University) and C57BL/6MRL.Fas^*lpr*^ mice were generous gifts from Jun Yan (University of Louisville). Lckcre mice and *Bim*^*fl/fl*^ mice were bought from Shanghai Model Organisms. Lckcre*Ube2m*^*fl/fl*^ mice (termed *Ube2m*^*-/-*^ mice) were generated by crossing Lckcre mice with *Ube2m*^*fl/fl*^ mice. Then these mice were crossed with C57BL/6MRL.*Fas*^*lpr*^ mice to acquire spontaneous lupus-prone mice with Ube2m deficiency (Lckcre*Ube2m*^*fl/fl*^MRL.*Fas*^*lpr*^, termed *Ube2m*^*-/-*^*lpr* mice) in T cells. Lckcre*Ube2m*^*+/+*^MRL.*Fas*^*lpr*^ or *Ube2m*^*fl/fl*^MRL.*Fas*^*lpr*^ mice were as control (termed WT*lpr* mice). Lckcre*Bim*^*fl/fl*^ mice (termed *Bim*^*-/-*^ mice) were generated by crossing Lckcre mice with *Bim*^*fl/fl*^ mice. Then these mice were crossed with C57BL/6MRL.*Fas*^*lpr*^ mice to acquire Lckcre*Bim*^*fl/fl*^MRL.*Fas*^*lpr*^mice, termed *Bim*^-/-^*lpr*. Lckcre*Ube2m*^*fl/fl*^*Bim*^*fl/fl*^MRL.*Fas*^*lpr*^ mice, termed *Ube2m*^*-/-*^*Bim*^*-/-*^*lpr*, were generated by crossing Lckcre*Ube2m*^*fl/fl*^MRL.*Fas*^*lpr*^ with *Bim*^*fl/fl*^MRL.*Fas*^*lpr*^ mice. Lckcre*Ube2m*^*+/+*^*Bim*^*+/+*^MRL.*Fas*^*lpr*^ or *Ube2m*^*fl/fl*^*Bim*^*fl/fl*^MRL.*Fas*^*lpr*^ mice were as control (collectively called WT*lpr* mice). Thymus index (thymus weight to mouse weight ratio) and the number of cells in thymus were calculated and T cell subsets in thymus were examined via flow cytometry from 8-week mice. At 8 months, the blood was obtained for flow cytometry and serum was collected. Subsequently, the mice were euthanized, and their kidneys and spleens were obtained for subsequent experimental analysis. For pristane-induced lupus, WT and *Ube2m*^*-/-*^ female mice (2 months) were administered with 0.5 ml of pristane or PBS via intraperitoneal injection. At 8 months, serum was collected and the mice were euthanized. Spleens and kidneys were obtained for following experiments.

All animal experiments in the study underwent review and were approved by the Institutional Animal Care and Use Committee of Zhejiang Chinese Medical University.

### Human samples

We collected peripheral blood samples with EDTA anticoagulant from normal and active SLE subjects. The patients met the classification criteria of the 2012 Systemic Lupus International Collaborating Clinics (SLICC) and were subjected to full history taking, thorough clinical and laboratory investigations.^55^ Then SLE disease activity index (SLE-DAI) was employed to determine the disease activity level. Healthy people were age and gender-matched individuals without underlying medical conditions. The research received approval from the Medical Ethics Committee of Zhejiang Chinese Medical University (2021-KL-1230-1) and consent informed consents have been provided freely by all participants.

### Detection of autoantibodies

The concentrations of total IgG and anti-dsDNA in serum were measured following the guidelines provided by the manufacturer (Multisciences Biotech Co., Ltd).

### The assessment of renal function

Fresh urine samples were manually harvested prior to sacrificing the mice. Then urine protein, albumin and creatinine were determined with corresponding kits following the instructions provided by Dia Sys Diagnostic Systems GmbH. The periodic acid-Schiff (PAS) and hematoxylin eosin (H&E) staining of renal histology as well as the pathological scores were performed according to our previous study.^56^

### Bio-Plex cytokine assay

Serum cytokines including IL-1β, IL-6, IL-10, IL-17, TNF-α and IFN-γ were measured using a Bio-Plex Pro Mouse Cytokine 6-plex panel (Bio-Rad) in accordance with the manufacturer’s protocols.

### FACS analysis

To explore the development of T cells in thymus, single-cell suspensions in thymus were prepared and subsequently stained with the following anti-mouse antibodies: anti-CD4 (PE-CY7), anti-CD8 (BV510), anti-CD25 (APC) and anti-CD44 (AF700). The proportion of T and B cells in mice was determined by staining splenic cells with anti-mouse antibodies: anti-CD3 (PE) and anti-CD19 (PB450) antibodies (Biolegend). To detect the CD3^+^ T cell subsets, splenic cells were stained with the indicated anti-mouse antibodies: anti-CD3 (PE), anti-CD4 (PE-CY7), anti-CD8 (APC) (Biolegend). To detect T cell percentage in human peripheral blood, anti-human CD3 (FITC), anti-human CD4 (PE-CY7) and anti-human CD8 (BV510) antibodies were used. All these stained cells were evaluated using Beckman CytoFlex S system (Beckman).

### Purification of DN T cells and T cells

DN T cells of spleens from mice were isolated using fluorescent cell sorting via BD FACSAria (BD Biosciences) by staining cells with anti-CD3 (PE), anti-CD4 (PE-CY7), anti-CD8 (APC) antibodies. CD4^+^ and CD8^+^ T cells from mice were obtained from spleens with corresponding Mouse T Cell Isolation Kits following the protocols provided by manufacturer (Stem Cell Technologies Inc). DN T cells of human peripheral blood were also isolated using a human DN T cell isolation kit (Miltenyi Biotec).

### 4D label free quantitative proteomic analysis

The 4D label-free quantitative proteomic analysis was conducted by Jingjie PTM BioLabs including protein extraction, trypsin digestion, HPLC fractionation, LC-MS/MS analysis, and bioinformatics analysis according to the method described previously.^57^

### Apoptosis assay

Annexin V/PI and Annexin V/7-AAD Staining Kit (Beyotime) were used to detect cell apoptosis. Splenic cells or peripheral blood in mice were stained with anti-CD3 (APC), anti-CD4 (PE-CY7) and anti-CD8 (BV510) antibodies (Biolegend), as well as Annexin V-PE and 7-AAD. Human peripheral blood was stained with anti-human CD3 (FITC), anti-human CD4 (PE-CY7) and anti-human CD8 (BV510) antibodies as well as Annexin V-PE and 7-AAD. Purified CD3^+^ T cells obtained from MRL/*lpr* mice were incubated with DMSO or MLN4924 (0.1 and 0.5 μM) for 12 h and then stained with the indicated antibodies: anti-CD3 (APC), anti-CD4 (PE-CY7) and anti-CD8 (BV510) (Biolegend). Peripheral Blood Mononuclear Cell (PBMC) isolated from patients were treated with 0.5 μM MLN4924 for 6 h and then stained with anti-human CD3 (APC-CY7), anti-human CD4 (PE-CY7) and anti-human CD8 (BV510) antibodies as well as Annexin V-FITC and PI-PE to detect cell apoptosis. All these cells were analyzed with Beckman CytoFlex S system (Beckman).

### Proliferation assays in vivo

The BeyoClick^TM^ EdU Cell Proliferation Kit with Alexa Fluor 488 (Beyotime) was employed for the detection of DN T cell proliferation. Mice were intraperitoneally administered with EdU (50 mg/kg) twice a day for 1 week according to previously described methods.^58^ Then spleen single-cell suspensions were stained with anti-mouse CD3 (APC), anti-mouse CD4 (BV510) and anti-mouse CD19 (PB450) antibodies (Biolegend). At last, the Click-iT reaction was carried out based on the protocols of manufacturer and cells were examined using Beckman CytoFlex S system (Beckman).

### Mitochondrial membrane potential (MMP) determination

The MMP in DN T cells was quantified using the JC-1 assay kit (Beyotime). Following the manufacturer’s recommendations, after surface staining with anti-mouse CD3 (APC-CY7), anti-mouse CD4 (PE-CY7) and anti-mouse CD8 (APC) antibodies, cells were incubated with JC-1 staining buffer at 37 °C for 20 min, and then analyzed by flow cytometry. The ratio of aggregates to monomers represents the change of MMP.

### Immunoblotting analysis

Cells were homogenized in RIPA buffer, containing phosphatase and protease inhibitors (Beyotime). The protein from cell lysate (40 μg) was processed with SDS-PAGE and transferred to nitrocellulose paper. Then the following proteins were detected with appropriate antibodies: Bim (Cell Signaling), Ube2m (Abcam), Ube2f (Proteintech), Cullin1 (Abcam), Caspase 3 (Cell Signaling Technology), Cleaved-caspase 3 (Cell Signaling Technology) and β-actin (Sigma-Aldrich). The band intensity was quantified with Image J software (NIH).

### Quantitative PCR analysis

The mRNA extraction, reverse transcription and real-time quantitative PCR were conducted in accordance with our previous article.^59^ The primer sequences were designed as follows: actin, sense 5’- GGCTGTATTCCCCTCCATCG-3’, antisense 5’- CCAGTTGGTAACAATGCCATGT −3’, Ube2m, sense 5’-AACCTGCCCAAGACGTGTG-3’, antisense 5’-AGCTGAATACAAACTTGCCACT-3’, Bim, sense 5’-CCCGGAGATACGGATTGCAC-3’, antisense 5’- GCCTCGCGGTAATCATTTGC −3’. The mRNA levels were determined with△△Ct method.

### Co-immunoprecipitation (Co-IP)

Cells were lysed in NP-40 buffer (Beyotime) supplemented with phosphatase and protease inhibitors, and the lysates were immunoprecipitated at 4 °C overnight with the SureBeads protein A (Bio-Rad) conjugated with the Bim antibody (Cell Signaling). Precipitates were washed three times with lysis buffer. Protein levels were evaluated using immunoblotting analysis with ubiquitin antibody (Thermo).

### Statistical analysis

The data were expressed as the mean ± SEM and analyzed with GraphPad Prism 8 software. Statistical significance was determined by *t*-test or two-way ANOVA, with *P*-values < 0.05 considered significant.

### Supplementary information

Supplementary_data-Neddylation-SLE

Author_Checklist

## Data Availability

The paper and Supplementary Materials contain all the necessary data for evaluating the conclusions. The proteomic data has been submitted to the ProteomeXchange Consortium via the PRIDE (Project accession: PXD045686).
