# Supplementary material for: Neddylation is a novel therapeutic target for lupus by regulating double negative T cell homeostasis
Source: Signal Transduct Target Ther. 2024 Jan 15;9:18. doi: 10.1038/s41392-023-01709-9 (PMC10788348; doi:10.1038/s41392-023-01709-9)

Supplementary Materials for

**Neddylation is a novel therapeutic target for lupus by** **regulating double negative T cell homeostasis**

Yun Zhang^1#^, Li-jun Du^1, 2#^, Chen-xi Wang^1#^, Zhang-sheng Jiang^1^, Qing-chi Duan^1^, Yi-ping Li^1^, Zhi-jun Xie^1^, Zhi-xing He^1^, Yi Sun^3,4^, Lin Huang^1^*, Li-wei Lu^5,6^*, Cheng-ping Wen^1^*

Correspondence to:  [chengpw2010@126.com](mailto:chengpw2010@126.com) (C.W.), [liweilu@hku.hk](mailto:liweilu@hku.hk) (L.L.), [huanglin@zcmu.edu.cn](mailto:huanglin@zcmu.edu.cn) (L.H.)

**This file includes:**

Supplementary Text

Figures. S1 to S8


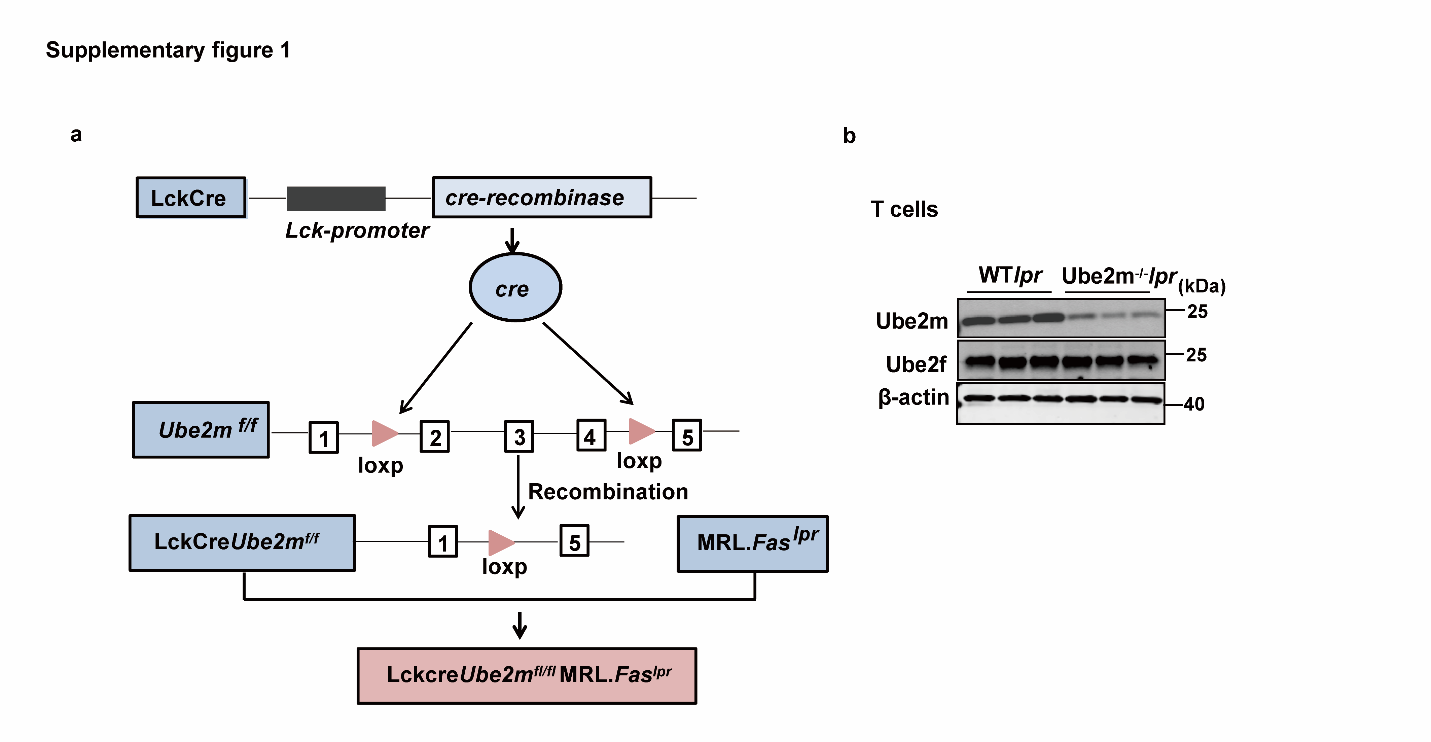


**Figure.S1. Generation of T cell-specific Ube2m knockout lupus-prone mice**

1. Schematic map of the generation of T cell Ube2m knockout lupus-prone mice. *Ube2m^f/f^* mice were crossed with Lckcre mice, in which the Ube2m floxed allele was detected and deleted by the cre recombinase expressed. Then Lckcre*Ube2m ^f/f^* mice were further crossed with MRL.*Fas^lpr^* to obtain T cell Ube2m knockout lupus-prone mice.
2. Immunoblotting analysis of Ube2m and Ube2f levels in T cells. Data were representative of three independent experiments.


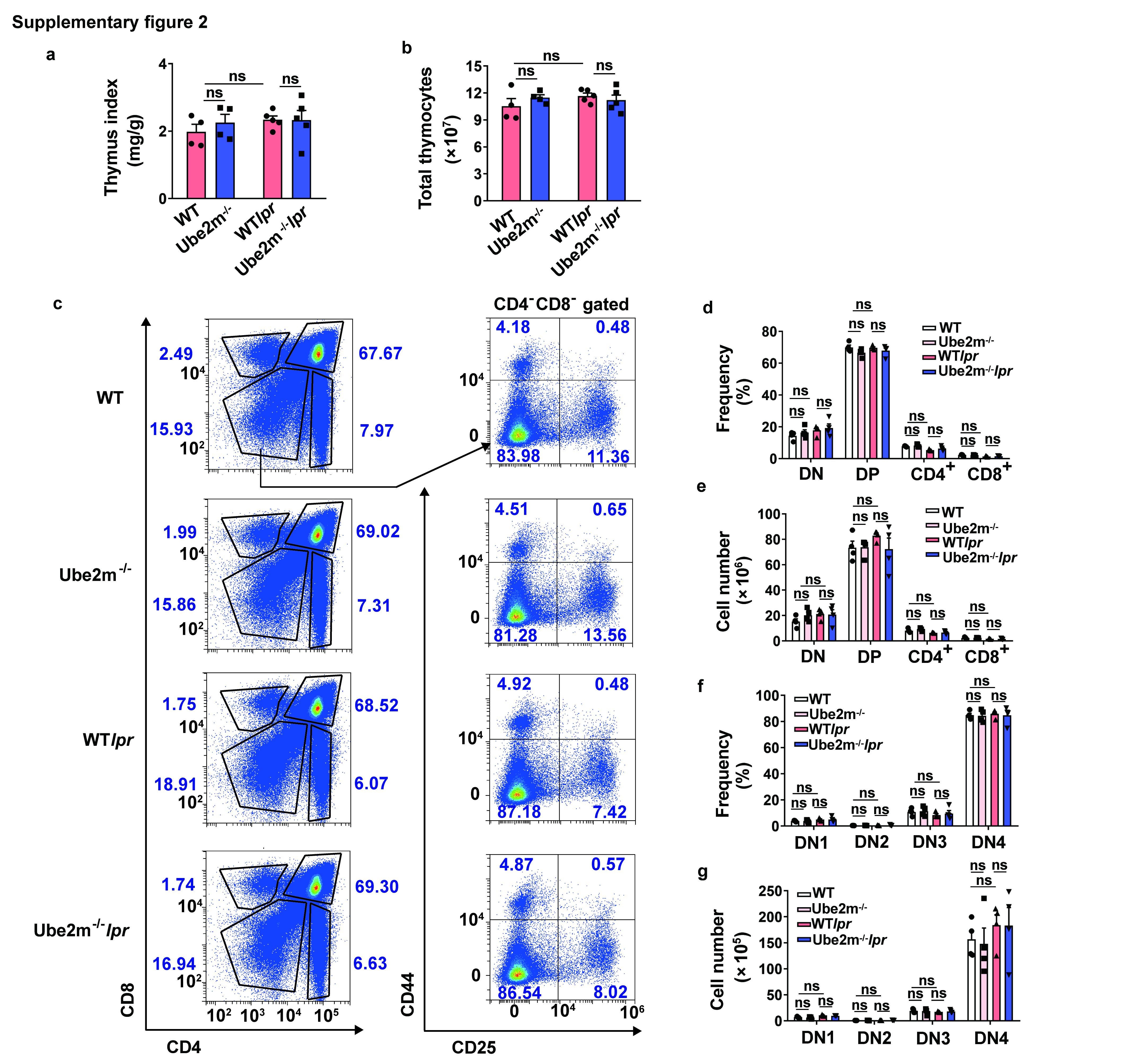


**Figure.S2. Characterization of T cell development in WT, Ube2m^-/-^, WT*lpr* and Ube2m^-/-^*lpr* mice**

a-b Thymus index (thymus weight to mouse weight ratios) and the number of cells in thymus from 8-week WT, Ube2m^-/-^, WT*lpr* and Ube2m^-/-^*lpr* mice. n= 4/group.

c-g Flow cytometric analysis and quantification of T cell subsets in thymus. n= 4/group.


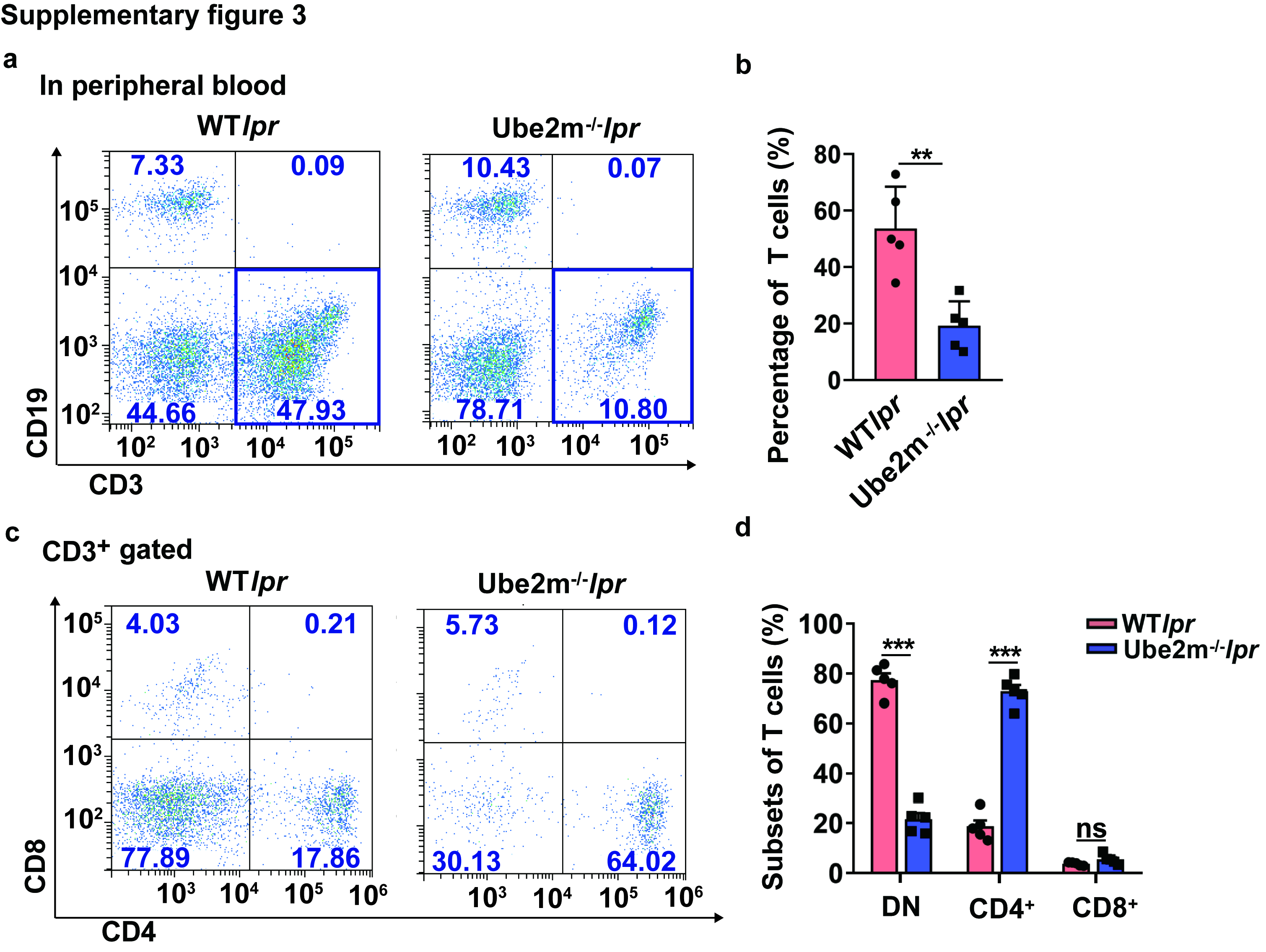


**Figure.S3. Loss of Ube2m prominently blocked T cell accumulation, especial DN T cell accumulation in peripheral blood**

a-b Proportion of T cells in peripheral blood was analyzed by flow cytometry and the percentage of T cells was quantified according to the results of flow cytometry. n= 5/group. ** *P* < 0.01.

c Flow cytometric analysis of CD3^+^ gated cells to identify T cell subsets including DN (CD4^-^CD8^-^), CD4^+^ and CD8^+^ T cells in peripheral blood. n= 5/group.

d The percentage of T cell subsets were quantified according to the results of flow cytometry. n= 5/group. *** *P* < 0.001.


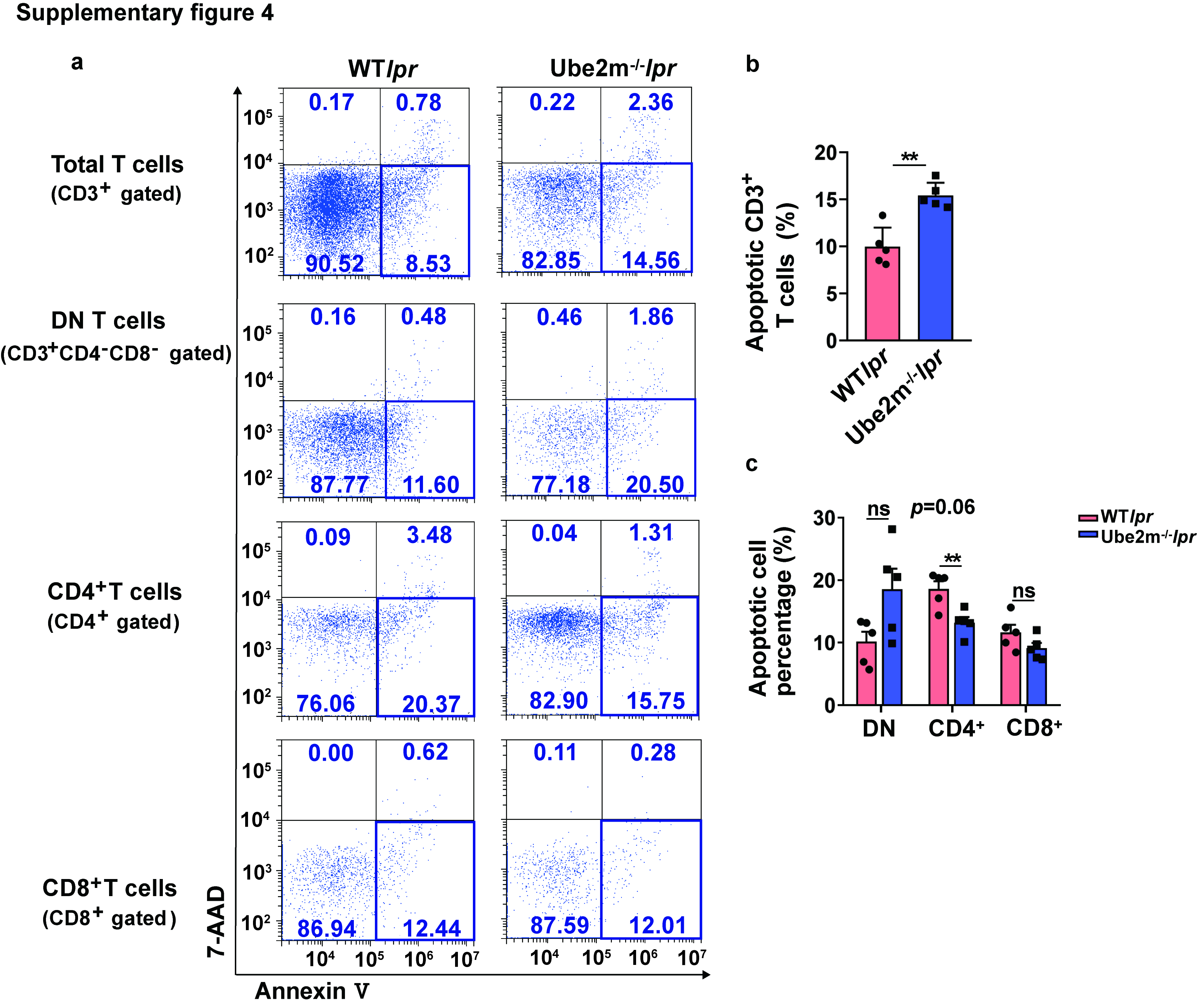


**Figure.S4. Increased apoptosis in Ube2m-KO DN T cells in peripheral blood**

1. The apoptosis (Annexin V^+^/7-AAD^-^) of T cells including total T cells, DN T cells, CD4^+^ and CD8^+^ T cells was evaluated with flow cytometry. n= 5/group.

b-c The apoptosis percentage of total T cells and T cell subsets was quantified according to the results of flow cytometry. n= 5/group. ** *P* < 0.01.


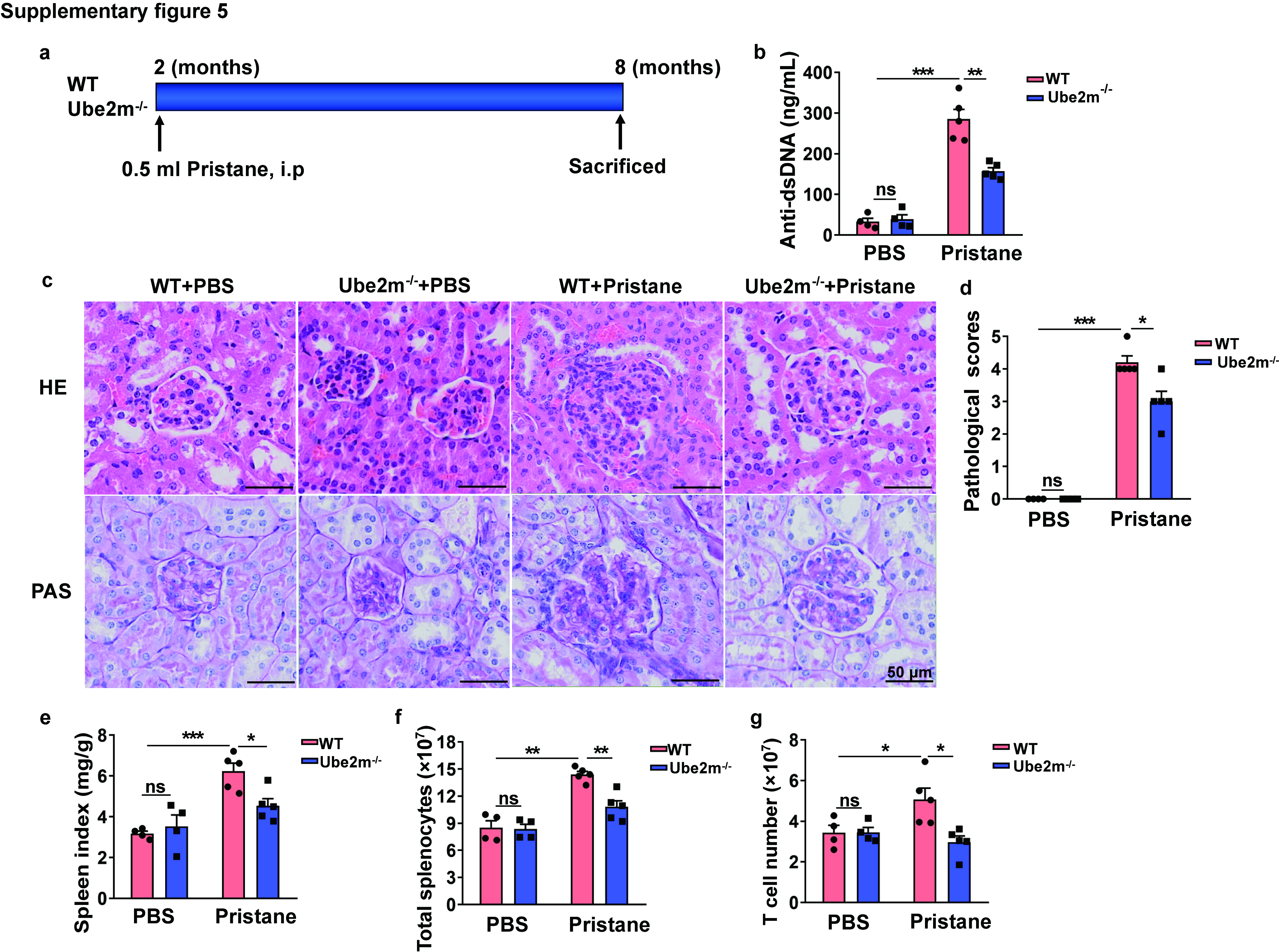


**Figure.S5. Loss of Ube2m attenuated lupus progression in pristane-induced lupus model**

1. WT and Ube2m^-/-^ female mice were randomly divided into two groups and administered with PBS or pristane (0.5 ml) via i.p for 6 months. n=4 (PBS groups) or n=8 (pristane groups).
2. The serum was collected at 8 months and then level of anti-dsDNA antibody was monitored. n= 4 (PBS groups) or n=5 (pristane groups). ** *P <* 0.01, *** *P* < 0.001.

c-d Representative images of PAS and HE staining from kidneys of 8-month-old mice were shown. Scale bar = 50 µm. Then, pathological score of each mouse was calculated. n= 4 (PBS groups) or n=5 (pristane groups). * *P* < 0.05, *** *P* < 0.001.

e-f Spleen index and the number of splenocytes were calculated. n= 4 (PBS groups) or n=5 (pristane groups). * *P* < 0.05, ** *P* < 0.01, *** *P* < 0.001.

g Statistical results of the T cell number. n= 4 (PBS groups) or n=5 (pristane groups). * *P <* 0.05.

**
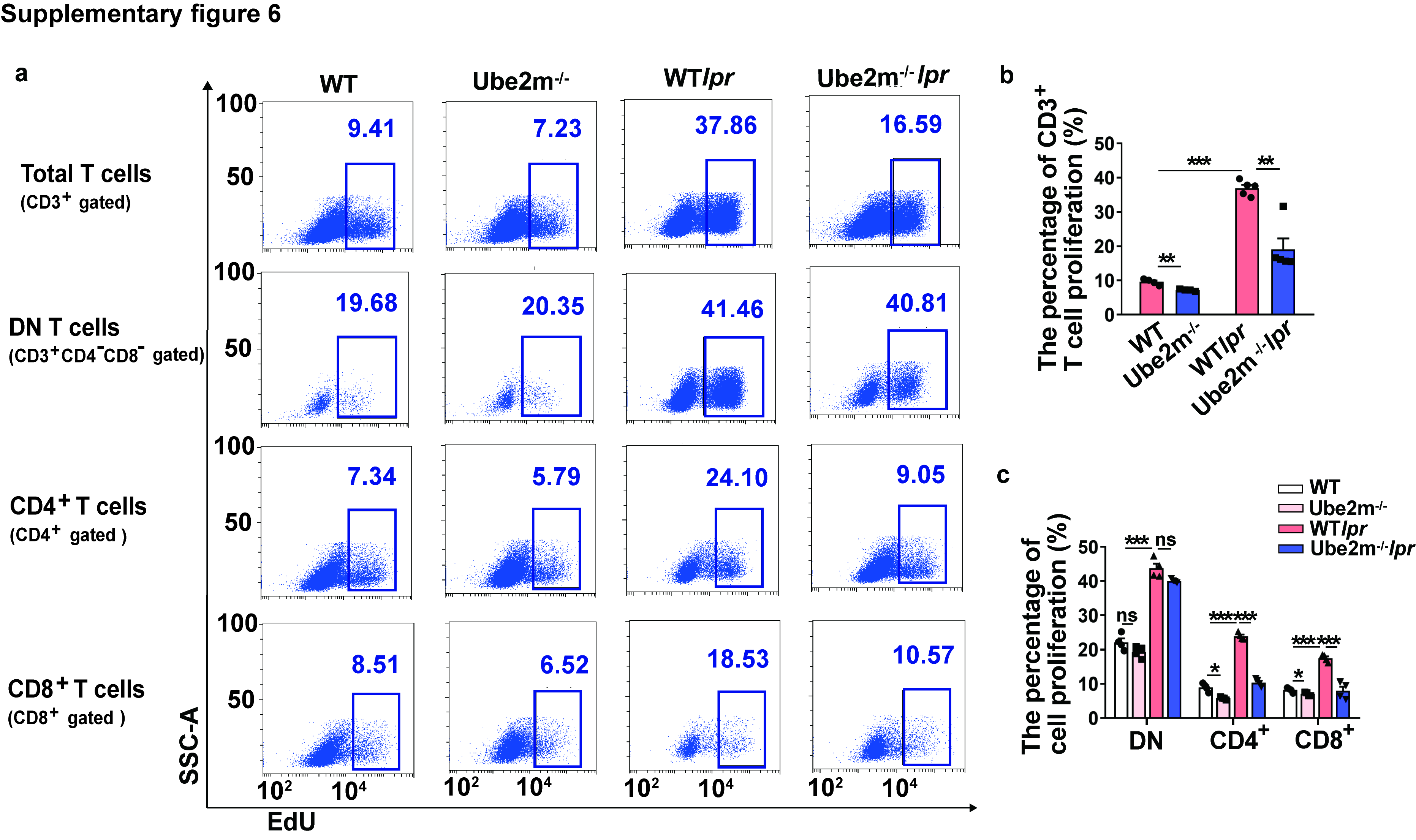
**

**Figure.S6. Normal proliferation of DN T cells for Ube2m deficiency**

a T cell proliferation (including total T cells, DN T cells, CD4^+^ and CD8^+^ T cells) of spleens was evaluated via EdU assay with flow cytometric analysis. n= 4 or 5/group.

b-c The proliferation percentage of total T cells and T cell subsets was quantified according to the results of flow cytometry. n= 4 or 5/group. ** *P* < 0.01, *** *P* < 0.001.

**
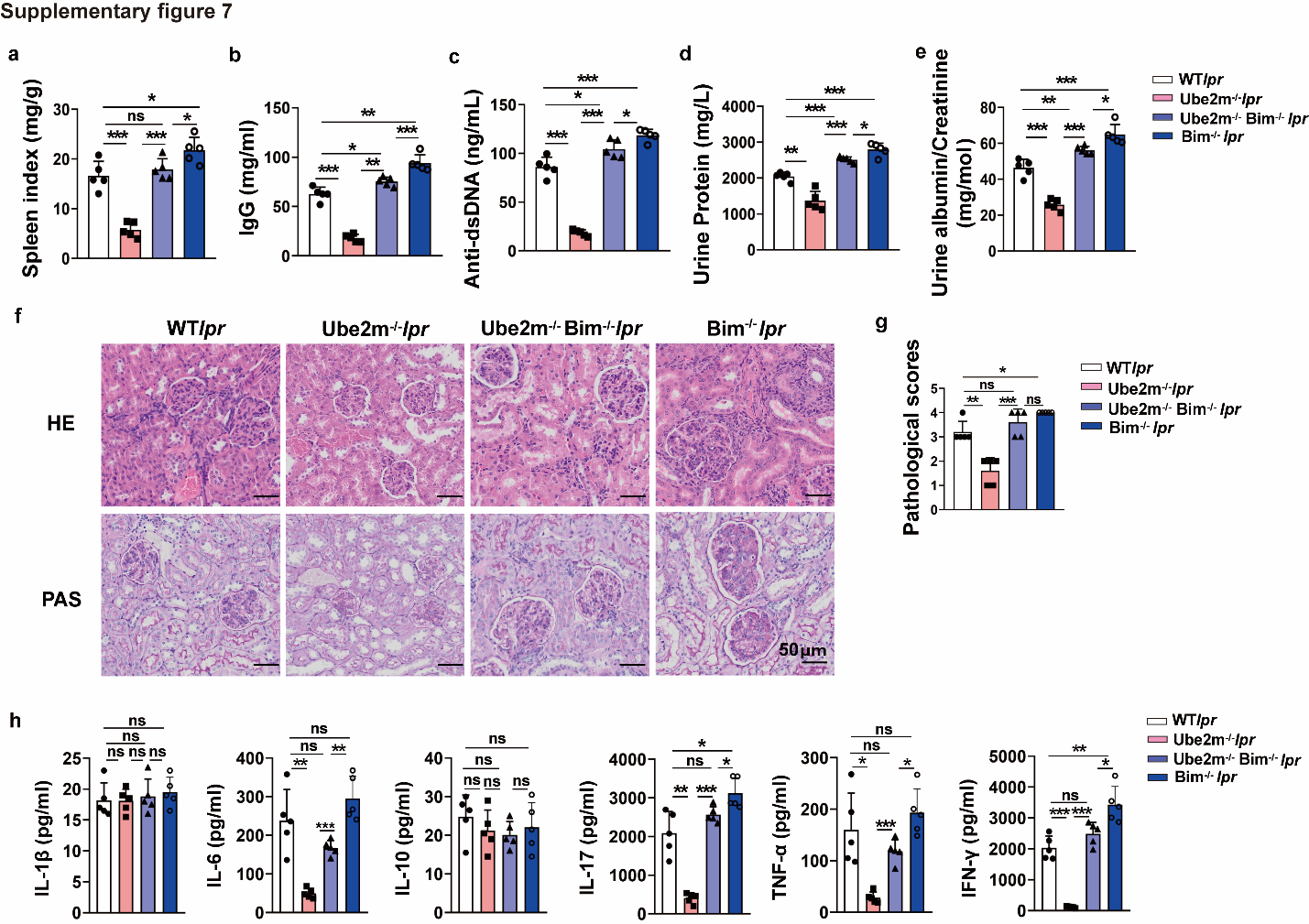
**

**Figure.S7. Loss of Bim reversed the alleviated lupus progression for Ube2m deficiency**

a Spleen index was calculated. n= 5/group. **P <* 0.05, *** *P* < 0.001.

b-c The level of IgG and anti-dsDNA antibodies in serum was measured. n= 5/group. * *P* < 0.05, ** *P* < 0.01, *** *P* < 0.001.

d-e Total protein, albumin and creatinine in urine were measured and the ratio of albumin to creatinine was calculated. n= 5/group. * *P* < 0.05, ** *P* < 0.01, *** *P* < 0.001.

f-g Representative images of PAS and HE staining from kidneys were shown. Then, pathological score of each mouse was calculated. Scale bar = 50 µm, n= 5/group. * *P* < 0.05, ** *P* < 0.01, *** *P* < 0.001.

h Cytokine level in serum was measured using Bio-Plex Pro^TM^ Mouse Cytokine Th17 panel A6-plex. n= 5/group. * *P* < 0.05, ** *P* < 0.01, *** *P* < 0.001.

**
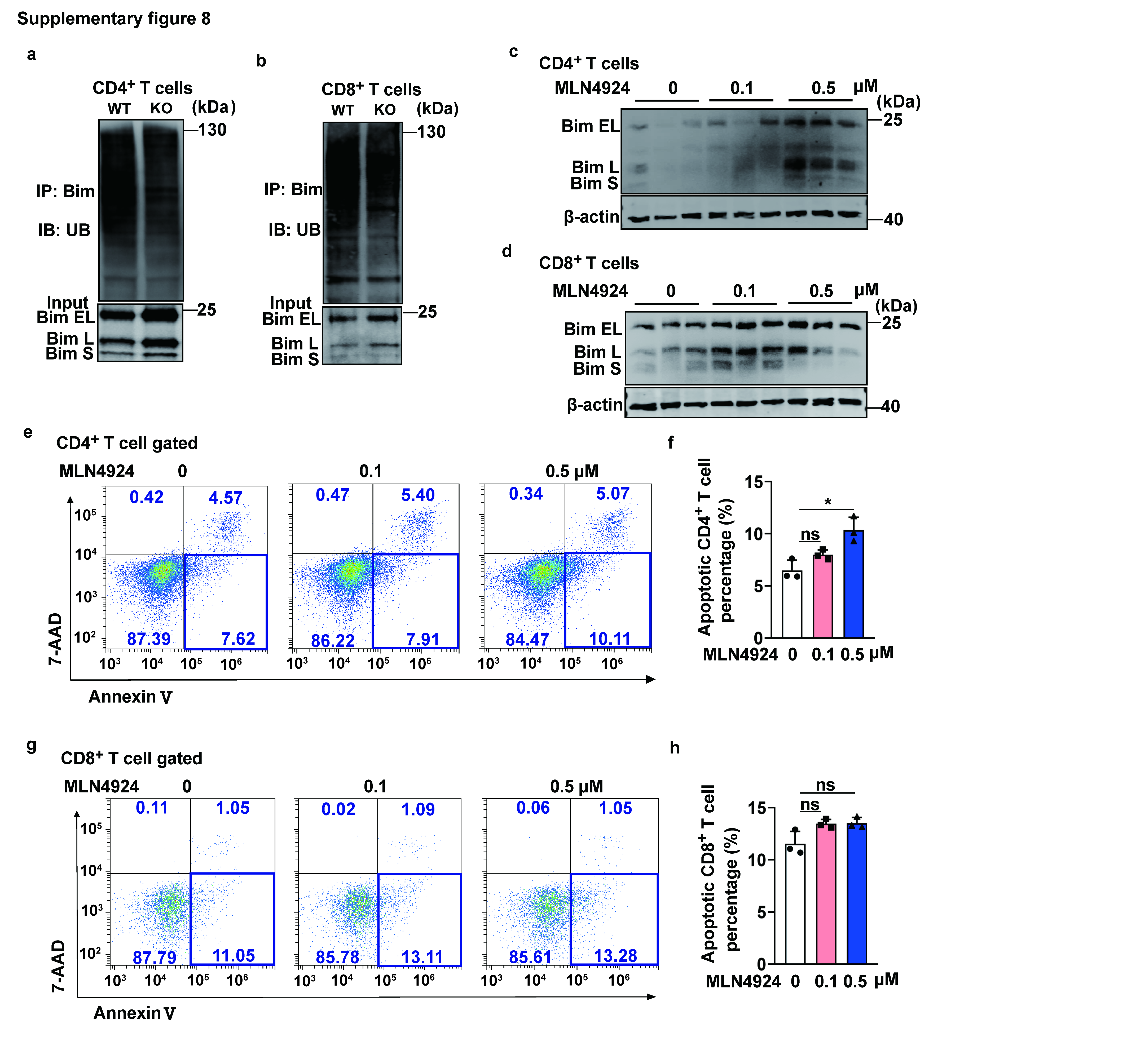
**

**Figure.S8. Neddylation inactivation upregulated Bim protein level in** **CD4^+^ and CD8^+^ T cells with less cell apoptosis compared with DN T cells**

a-b Ubiquitination degradation of Bim in CD4^+^ and CD8^+^ T cells from WT*lpr* and Ube2m^-/-^*lpr* mice was measured via Co-IP assay. Data were representative of three independent experiments.

c-d Immunoblotting assay showed the level of Bim in MLN4924 treated-CD4^+^ and CD8^+^ T cells.

e The apoptosis of CD4^+^ T cells treated with MLN4924 were detected via flow cytometry (Annexin V^+^/7-AAD^-^).

f The apoptosis percentage of CD4^+^ T cells treated with MLN4924 was quantified according to the results of flow cytometry. n= 3/group. * *P <* 0.05.

g The apoptosis of CD8^+^ T cells treated with MLN4924 were detected via flow cytometry (Annexin V^+^/7-AAD^-^).

h The apoptosis percentage of CD8^+^ T cells treated with MLN4924 was quantified according to the results of flow cytometry. n= 3/group.

**Other Supplementary Materials for this manuscript include the following:**

original and uncropped films of Western blots


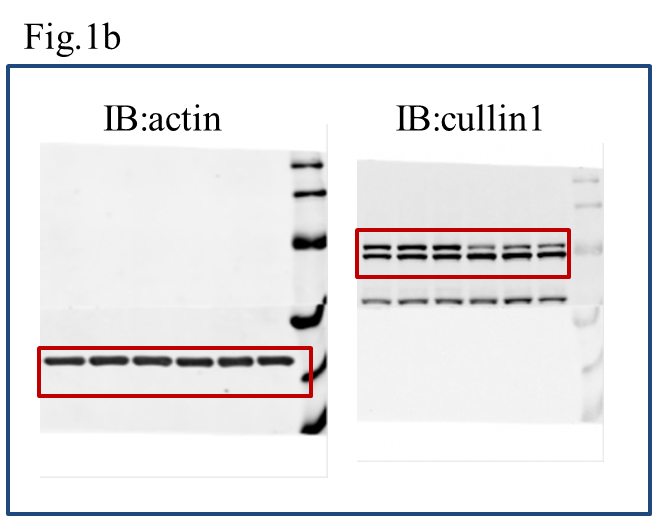


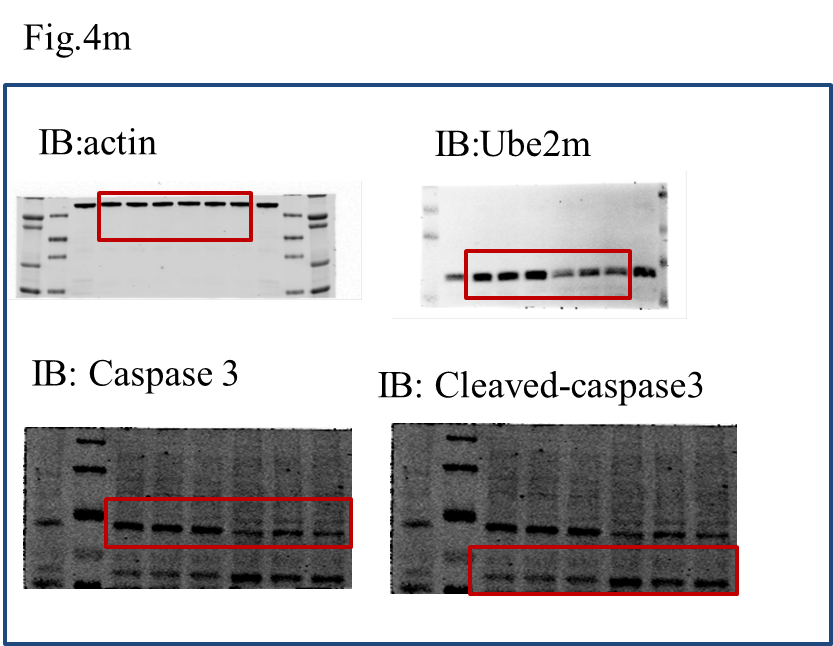


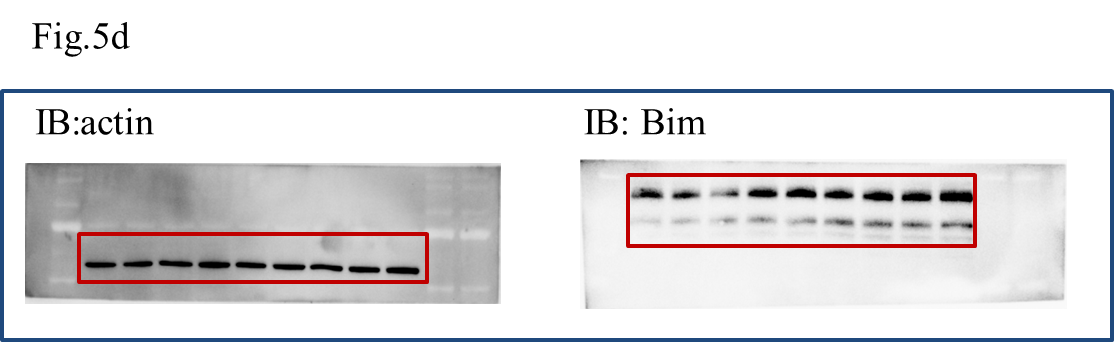


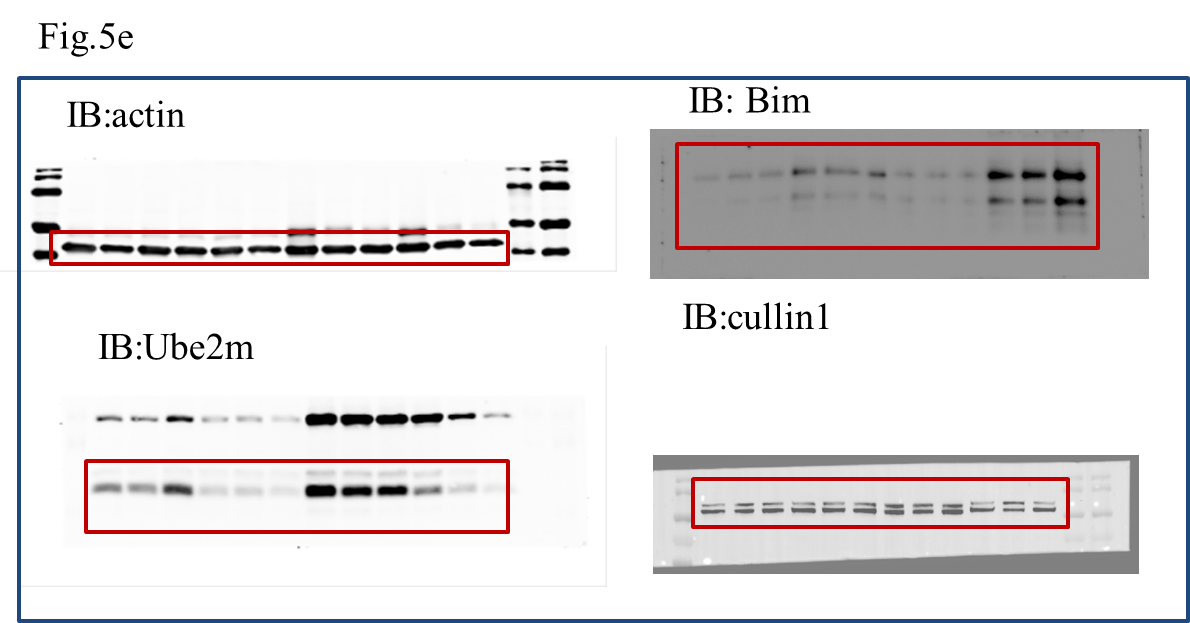


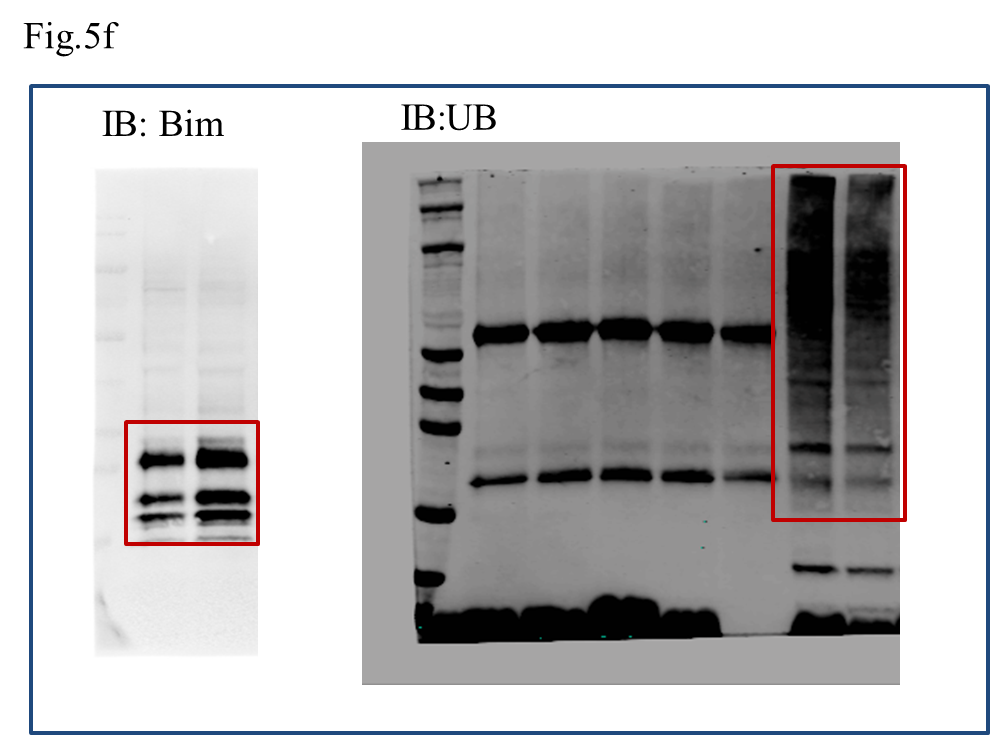


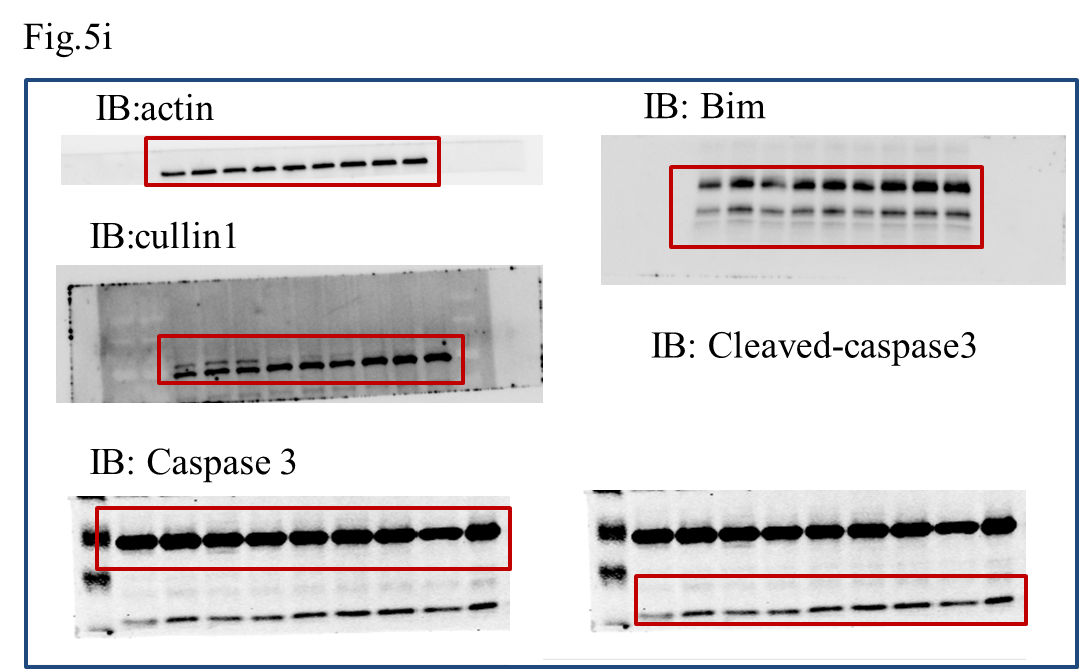


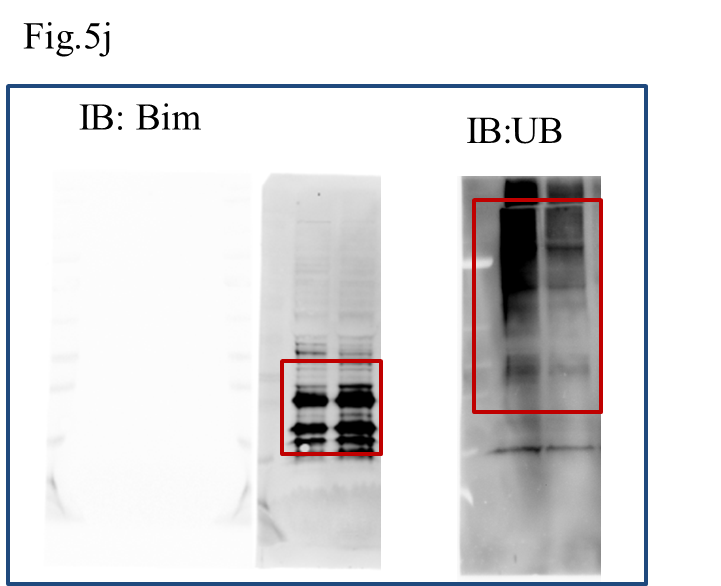


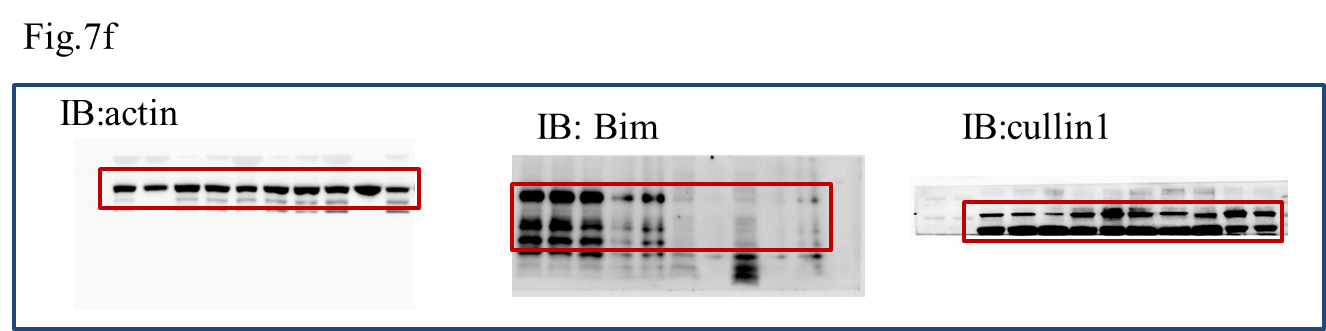


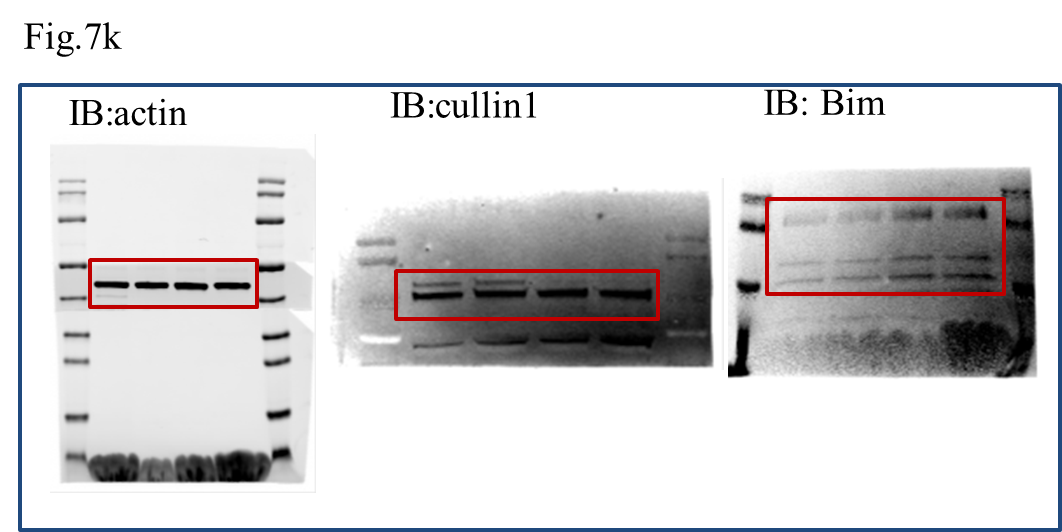


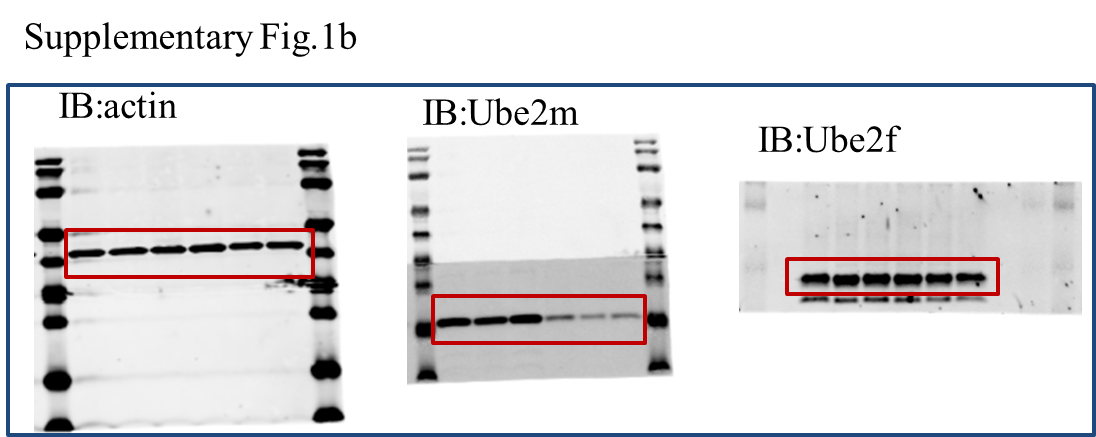


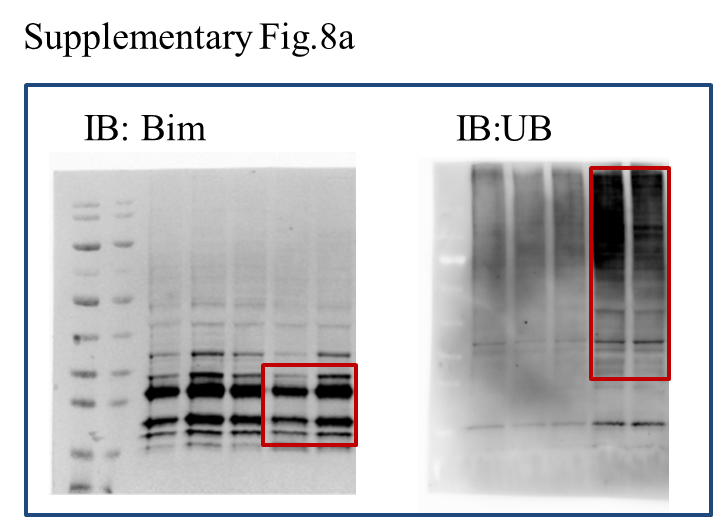


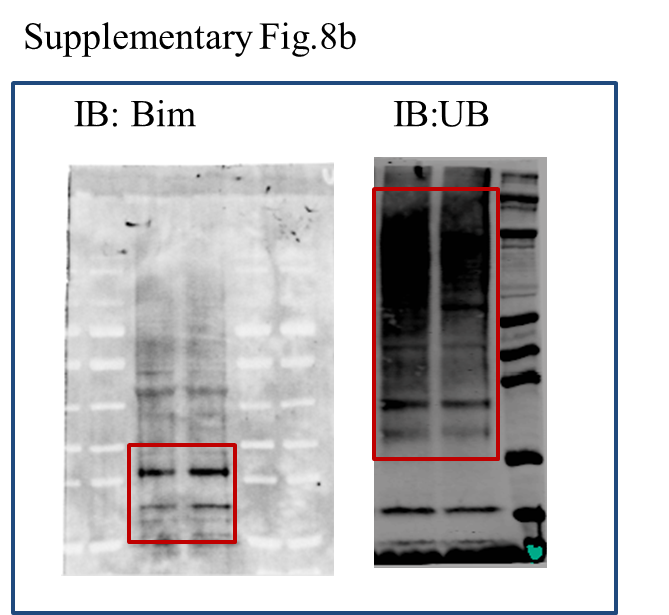


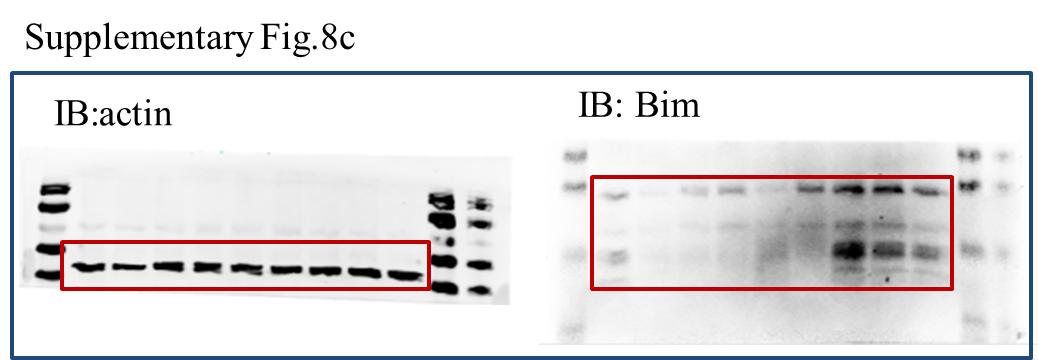


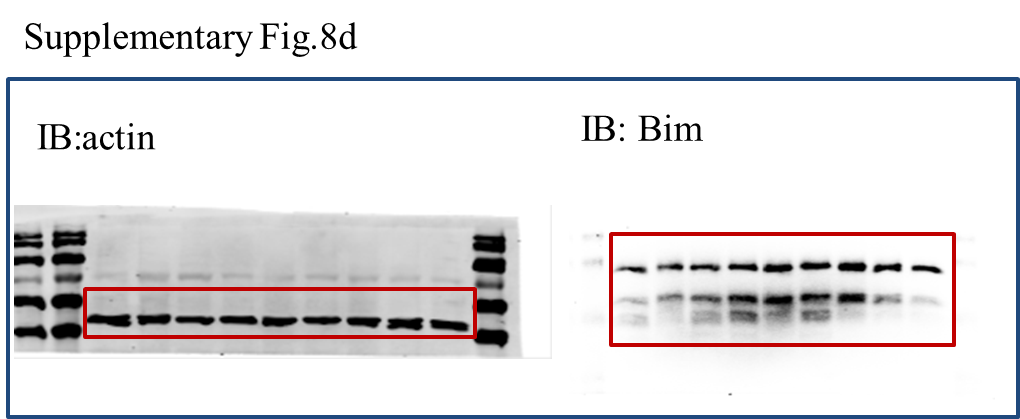

Supplement: Supplementary file 1 — Supplementary_data-Neddylation-SLE [file 41392_2023_1709_MOESM1_ESM.docx]
